# Effect of micronutrients on the risk of Graves’ disease: a Mendelian randomization study

**DOI:** 10.3389/fnut.2024.1432420

**Published:** 2024-12-09

**Authors:** Fangsen Chen, Rongliang Qiu, Zhiqing Lin, Junhan Chen, Peitian Liu, Yanling Huang

**Affiliations:** ^1^Department of Endocrinology and Metabolism, Zhongshan Hospital of Xiamen University, School of Medicine, Xiamen University, Xiamen, China; ^2^The School of Clinical Medicine, Fujian Medical University, Fuzhou, China; ^3^Department of General Surgery, Zhongshan Hospital, Xiamen University, Xiamen, China; ^4^Department of Pediatrics, Zhongshan Hospital of Xiamen University, School of Medicine, Xiamen University, Xiamen, China

**Keywords:** Mendelian randomization, Graves’ disease, micronutrients, copper, iron, zinc, calcium, vitamins

## Abstract

**Background:**

Micronutrient research on Graves’ disease (GD) is limited and controversial. Therefore, in order to explore possible correlations between genetically predicted amounts of six micronutrients [Copper (Cu), Iron (Ir), Zinc (Zn), Calcium (Ca), Vitamin C (VC), and Vitamin D (VD)] and GD risk, we carried out Mendelian randomization research (MR).

**Methods:**

We conducted an MR analysis using genome-wide association studies (GWAS) from people of European ancestry and aggregated information from UK Biobank to provide insight into the relationships between micronutrients and GD. The causal link between exposure and outcome was tested using three different techniques: Inverse Variance Weighted (IVW), MR-Egger, and Weighted Median Estimator (WME). The heterogeneity of outcomes was also assessed using Cochran’s Q statistic, and pleiotropy was assessed by MR-Egger intercept, MR-PRESSO.

**Results:**

IVW analyses showed evidence of no significant effect of genetically predicted micronutrient concentrations on GD, except for Cu. (Cu: OR = 1.183, *p* = 0.025; Ir: OR = 1.031, *p* = 0.794; Zn: OR = 1.072, *p* = 0.426; Ca: OR = 1.040, *p* = 0.679; VC: OR = 1.011, *p* = 0.491; VD: OR = 0.902, *p* = 0.436). Significant heterogeneity was observed in Ca and VD (Ca: Q = 264.2, *p* = 0.002; VD: Q = 141.42, *p* = 0.047). The MR-Egger intercept method identified horizontal pleiotropy between serum Ca levels and GD (MR-Egger intercept = −0.010, *p* = 0.030), with no similar findings for other micronutrients.

**Conclusion:**

MR analysis showed a possible causal relationship between the genetically predicted concentration of Cu and the risk of GD, whereas the genetically predicted concentrations of Ir, Zn, Ca, VC, and VD may not be causally related to the risk of GD.

## 1 Introduction

Graves’ disease (GD), an autoimmune thyroid disease (AITD), is the most prevalent cause of hyperthyroidism, potentially resulting in goiter, protruding eyeballs, pretibial mucous edema, and an elevated metabolic state, all of which pose significant health risks (1, 2). Thyroid Stimulating Hormone Receptor Antibody (TRAb) is the primary immunological characteristic of GD (2, 3). Risk factors for GD include genetic predisposition, environmental factors, and immunological factors (4). There is now considerable evidence suggesting the potential significance of micronutrients in preventing and mitigating GD, indicating that they may play a pivotal role in thyroid physiology and pathology (5, 6). Micronutrients may play a crucial role in the thyroid’s physiological and pathological activities.

Research has revealed the importance of critical metals for several typical physiological processes and activities, including copper (Cu), iron (Ir), zinc (Zn), and calcium (Ca). Variations in the levels of these essential metals have the potential to affect the risk of thyroid disease, including hyperthyroidism (7). Previous studies have also shown that antioxidant supplementation (vitamins C and E, beta-carotene, and selenium) in treating GD results in faster normalization of thyroid function (8). Furthermore, certain studies have postulated that decreased vitamin D levels may be connected to an increased risk of Graves’ disease (9). Nonetheless, there is a scarcity of clear data regarding the impact of vitamin D levels on thyroid function in healthy individuals (10).

Cu, being one of the most abundant minerals in the body, plays a pivotal role in the functioning and maintenance of the immune system. During thyroid metabolism, particularly in the processes of hormone production and absorption, Cu exerts an influence on the production and absorption of Thyroxine (T4) by modulating Ca levels within the body (11). Concerning the association between serum Cu and thyroid autoimmunity, existing research findings appear to be inconsistent. A positive correlation has been reported between elevated serum Cu concentrations and thyroid autoantibody presence in some studies (12). Nonetheless, another study observed no significant association between Cu levels and thyroid autoimmune inflammation or thyroid autoantibodies (13).

Ir, an essential element for human health, plays a pivotal role in redox reactions and oxygen transportation in the body. Iron homeostasis is closely related to thyroid function, with studies finding that serum iron levels positively correlate with Free Triiodothyronine (FT3) and Free Thyroxine (FT4) levels (14). Nevertheless, literature reports also indicate a positive correlation between iron and FT3, but not with FT4, with the most significant positive correlation observed in the iron-to-FT3:FT4 ratio (7). At present, the research on the relationship between Ir and GD is not enough.

Zn is indispensable to human health, and numerous autoimmune diseases are closely correlated with pathological alterations in Zn levels. This correlation significantly impacts the proper orchestration of signaling, leading to profound changes in immune response, cell differentiation, and functionality (15). A cross-sectional study has demonstrated a notable association between serum Zn concentrations and thyroid volume in patients suffering from nodular goiter. Additionally, a similar significant correlation was identified between serum Zn levels and thyroid autoantibody levels among patients diagnosed with AITD. Notably, even among individuals with normal thyroid function, a significant correlation was evident between serum Zn levels and FT3 concentrations (16).

Ca ions are well-established second messengers integral to a diverse array of signal transduction processes (17). The frequency of hypercalcemia among those with hyperthyroidism is 38% (18). A study in rats found that hyperthyroidism leads to structural and functional changes in the hepatic mitochondrial calcium transport system, thereby affecting calcium accumulation and retention capabilities (19). Observational studies have not uncovered a direct correlation between Ca and GD.

VC is integral to the cellular functions of both the innate and adaptive immune systems. Its antioxidant properties function as cofactors for a range of biosynthetic and gene-regulating enzymes, critically contributing to immunomodulatory mechanisms. These mechanisms encompass neutrophil migration to infection sites, augmented phagocytosis, oxidant generation, and microbial elimination (20). Prior research has investigated the level of VC in patients with thyroid disease and its potential influence on thyroid drug absorption. Nonetheless, the available evidence pertaining to the association between plasma VC, GD, and GD therapy remains limited.

VD primarily serves as a regulator of mineral homeostasis. Notably, recent studies have established a strong correlation between insufficient VD levels and AITD, particularly Hashimoto’s thyroiditis (HT) and GD (21, 22). Currently, the causality between VD deficiency and AITD remains ambiguous; specifically, it is uncertain whether VD deficiency is a trigger for AITD or if thyroid dysfunction leads to lower VD levels (23).

The synthesis and metabolism of thyroid hormones rely on a diverse array of micronutrients for maintaining normal thyroid function. These micronutrients coexist in a state of dynamic equilibrium, wherein their interactions are crucial. However, this delicate balance can be perturbed by an imbalance in the quantity of one or multiple components, potentially leading to dysfunction of the thyroid gland and a heightened susceptibility to autoimmune thyroid disorders (24, 25).

The causal relationship between micronutrients and thyroid disease is not yet clear, but the potential application of micronutrients in the correction of GD disease is gaining attention. Although various studies have established associations between multiple micronutrients and GD, the problem of possible bias caused by confounding factors still cannot be ignored. In addition, residual confounding and reverse causality present in traditional observational studies pose challenges in quantifying causal effects. Rigorously designed randomized controlled trials (RCTs), acknowledged as the gold standard for causal inference, aid in minimizing the impact of potential confounders. However, ethical considerations, external validity limitations, challenges in double-blinding, and interference from internal and external factors, coupled with inadequate statistical power and significant financial/temporal requirements, have hampered the feasibility of conducting RCTs.

Mendelian Randomization (MR) serves as an epidemiological approach for investigating causal links between exposure factors and outcome variables. By harnessing genetic randomization, MR efficiently circumvents potential confounding factors (26). MR analyses leverage single nucleotide polymorphism (SNP) data from independent Genome-Wide Association Studies (GWAS) to conduct SNP-exposure and SNP-outcome association analyses, ultimately yielding causal effect estimates (27). Employing a two-sample MR genetic prediction approach, this study aimed to elucidate potential causal associations between exposures to Cu, Ir, Zn, Ca, VC, VD, and GD, thereby enhancing our understanding of the relationship between micronutrients and GD.

## 2 Materials and methods

### 2.1 Research design

**Figure 1:**
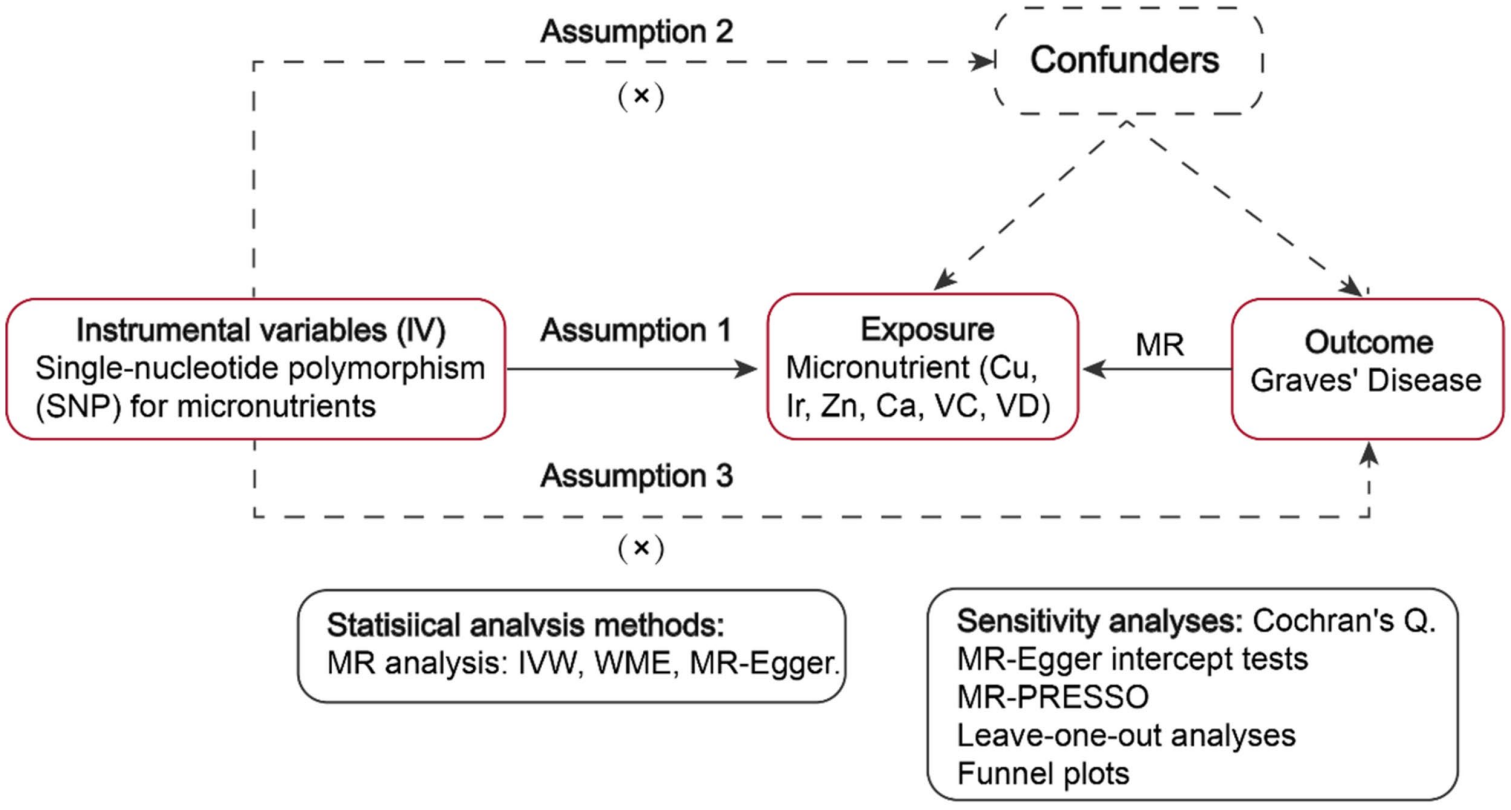
Workflow of the MR study demonstrating the link between Graves’ disease and micronutrients. IVW, inverse variance weighted; MR, Mendelian randomization; WME, weighted median, SNP, single-nucleotide polymorphisms.

GWAS summary statistics about six micronutrients (including Cu, Ir, Zn, Ca, VC, and VD) as well as Graves’ disease were retrieved from publicly accessible data repositories. Subsequently, MR analyses were employed to assess genetic causality (Figure 1).

### 2.2 Determination of the instrumental variables

Instrumental variables (IVs) are characterized by three pivotal assumptions: (1) Association assumption: There exists a robust correlation between SNPs and exposure factors. (2) Independence assumption: The SNP remains unaffected by confounding factors. (3) Exclusivity assumption: The SNP solely influences the outcome via exposure factors (27).

GWAS pooled data for Cu, Ir, Zn, Ca, VC, and VD were screened for statistically significant SNPs (*p* < 5 × 10^8^, LD R^2^ < 0.001, genetic distance = 10,000 KB). To uphold the initial hypothesis of Mendelian Randomization, requiring a strong correlation between IVs and exposure, weak IVs were identified by calculating the F-statistic (F = bet^2^/se^2^) for each SNP. An *F*-value greater than 10 suggests the absence of weak IVs (28). As the SNPs employed in MR adhere to the principle of random allele transmission from parents to offspring and are unlikely to be subject to the environment, it is plausible to assume that IVs are independent of confounders, thereby fulfilling the independence assumption of MR (27).

Furthermore, the study examined horizontal pleiotropy using MR-Egger intercept analysis. A *p*-value above 0.05 suggests no horizontal pleiotropy, validating the exclusivity assumption and confirming the IVs sole association with the outcome via the exposure factor (29).

### 2.3 Data extraction

In this study, we searched Open GWAS for statistical summary data related to micronutrient cycling concentrations as exposure. For Cu circulating concentrations, GWAS summary data encompassed 2,603 European individuals, revealing 2 SNPs strongly associated with Cu levels (Supplementary Table S1). In the case of Ir circulating concentrations, the dataset comprised 23,986 individuals of European ancestry, identifying 3 significant SNPs (Supplementary Table S2). Regarding Zn circulating concentrations, data encompassed 2,603 European individuals, identifying 2 significant SNPs (Supplementary Table S3). Ca circulating concentrations were assessed in 315,153 individuals of European descent, uncovering 212 significant SNPs (Supplementary Table S4). VC circulating concentrations were examined in 291 European individuals, revealing 68 significant SNPs (Supplementary Table S5). Finally, VD circulating concentrations were investigated in 496,946 European individuals, identifying 117 significant SNPs (Supplementary Table S6). As for outcomes, GWAS summary statistics for GD included 458,620 European individuals, sourced from the UK Biobank database.

### 2.4 MR analyses

We utilized three MR methods: Inverse Variance Weighted (IVW), MR-Egger, and Weighted Median Estimator (WME), to evaluate the causal link between micronutrients and GD risk. IVW, the main analytical method, provides unbiased causal estimates without horizontal pleiotropy and is considered the most informative. Complementing IVW, MR-Egger, and the WME offer more robust estimates under less stringent conditions. MR-Egger is effective in yielding causal estimates in the presence of some horizontal pleiotropy, while WME has a lower sensitivity to outliers and measurement errors (30, 31).

### 2.5 Sensitivity analyses

Heterogeneity was evaluated via Cochran’s Q for the IVW method, indicating statistical significance at *p* < 0.05 (32). Horizontal pleiotropy was assessed using the MR-Egger intercept method and the MR-PRESSO technique, with significance set at *p* < 0.05 (29). MR-PRESSO is comprised of three components: (1) Identification of horizontal pleiotropy, (2) Correction of horizontal pleiotropy via outlier elimination, and (3) Analysis of causal estimates, both before and following outlier correction, to ascertain the presence of any disparities. The leave-one-out methodology was implemented to evaluate the impact of specific SNPs on the outcomes of MR analyses (32).

The MR analyses were performed using the R packages “TwoSampleMR” and “MRPRESSO” in the R software environment (v4.1.1).

## 3 Results

### 3.1 Selection of genetic instrumental variables

Following stringent quality control measures, six genetic instruments were developed for the current study. These include: (1) Related datasets of exposure Cu and outcome GD were combined, encompassing 2 SNPs for analysis. (2) Datasets of exposure Ir and outcome GD were combined, including 3 SNPs for analysis. (3) The combination of exposure Zn and outcome GD datasets encompassed 2 SNPs for analysis. (4) Following the integration of exposure Ca and outcome GD datasets and the removal of eight palindromic sequences (rs12626330, rs1763519, rs296849, rs35095338, rs4517550, rs4744854, rs490275, rs7839633), 204 SNPs were ultimately included for analysis. (5) The integration of exposure VC and outcome GD datasets yielded 68 SNPs for subsequent analysis. (6) Upon the combination of exposure VD and outcome GD datasets, with the exclusion of one palindromic sequence (rs7955128), 116 SNPs were ultimately incorporated for analysis.

### 3.2 MR analysis results

A two-sample MR study investigated the causal relationships between SNPs associated with Cu, Ir, Zn, Ca, VC, and VD and the outcome of interest, GD, using genetic data from European populations. To achieve this, three methods were employed to estimate instrumental variables, namely IVW, WME, and MR-Egger (Table 1).

1. A significant causal relationship was observed between Cu levels and GD, as indicated by the IVW analysis (OR 1.183, 95% CI 1.020–1.372, *p* = 0.025).
2. No significant causal relationship was detected between Ir levels and GD using IVW (OR 1.031, 95% CI 0.815–1.305, *p* = 0.794), MR-Egger (OR 1.043, 95% CI 0.503–2.162, *p* = 0.927), and WME (OR 1.027, 95% CI 0.835–1.263, *p* = 0.796).
3. No significant causal association was found between Zn levels and GD, according to the IVW analysis (OR 1.072, 95% CI 0.903–1.272, *p* = 0.426).
4. No significant causal relationship was observed between Ca levels and GD, despite the MR-Egger (OR 1.489, 95% CI 1.026–2.162, *p* = 0.037) and WME (OR 1.419, 95% CI 1.069–1.884, *p* = 0.015) analyses indicating potential significance. However, the IVW analysis (OR 1.040, 95% CI 0.861–1.256, *p* = 0.679) did not support this finding.
5. No significant causal relationship was observed between VC levels and GD, as evidenced by the IVW (OR 1.011, 95% CI 0.978–1.045, *p* = 0.491), MR-Egger (OR 1.021, 95% CI 0.947–1.101, *p* = 0.580), and WME (OR 0.993, 95% CI 0.946–1.044, *p* = 0.809) analyses.
6. No significant causal association was detected between VD levels and GD, according to the IVW (OR 0.902, 95% CI 0.747–1.133, *p* = 0.436), MR-Egger (OR 1.127, 95% CI 0.817–1.555, *p* = 0.465), and WME (OR 1.231, 95% CI 0.915–1.656, *p* = 0.169) assessments.

**Table 1:** Mendelian randomization estimates for the association between Graves’ disease and micronutrient.

Exposure	Outcome	n SNPs	IVW OR (95% CI)	IVW *p*-value^a^	Cochrane’s Q	Cochrane’s Q *p*-value^a^	MR-Egger Intercept^b^	MR-Egger Intercept *p*-value^a^
Copper	Graves’ disease	2	1.183 (1.020–1.372)	0.025	0.043	0.835	NA^C^	NA^c^
Iron	Graves’ disease	3	1.031 (0.815–1.305)	0.794	2.980	0.225	−0.001	0.987
Zinc	Graves’ disease	2	1.072 (0.903–1.272)	0.426	1.632	0.201	NA^C^	NA^C^
Calcium	Graves’ disease	204	1.040 (0.861–1.256)	0.679	264.2	0.002	−0.010	0.030
Vitamin C	Graves’ disease	68	1.011 (0.978–1.045)	0.491	141.42	0.047	−0.007	0.108
Vitamin D	Graves’ disease	116	0.902 (0.747–1.133)	0.436	58.602	0.757	−0.005	0.776

a Nominal *p*-value.

b The MR-Egger intercept quantifies the effect of directional pleiotropy (*p* < 0.05, which means possible pleiotropy).

c Insufficient number of SNPs for MR-Egger analysis.

We found little evidence that genetically predicted Ir, Zn, Ca, VC, and VD concentrations other than Cu had a significant effect on GD risk.

### 3.3 Sensitivity analysis results

In the sensitivity analysis phase, the heterogeneity of various micronutrient-related SNPs concerning GD impact studies was initially evaluated using Cochran’s Q test. The test revealed that, except for Ca and VD, SNPs associated with other micronutrients, including Cu, Ir, Zn and VC, did not exhibit significant heterogeneity. The specific results are as follows: Cu: Q = 0.043, *p* = 0.835; Ir: Q = 2.980, *p* = 0.225; Zn: Q = 1.632, *p* = 0.201; Ca: Q = 264.2, *p* = 0.002; VD: Q = 141.42, *p* = 0.047; VC: Q = 58.602, *p* = 0.757. Despite the observed heterogeneity in certain micronutrients, we have accounted for this to some extent by using the IVW random effects model for our analysis. Therefore, our conclusions are still based on our primary analysis method, the IVW method.

Utilizing the MR-Egger intercept method, we assessed the extent of horizontal pleiotropy and observed a significant association between serum Ca and the outcome of interest, GD (MR-Egger intercept = −0.010, *p* = 0.030). However, the outlier value (rs7108820) was subsequently excluded via the application of the MR-PRESSO technique. A statistical test was performed after the level of pleiotropy was corrected to ascertain the significance of the difference between the causal estimations before and after the correction (*p* = 0.815), further disproving a causal association between Ca and GD. *p*-values obtained using the MR-Egger intercept approach for the other micronutrients were consistently above 0.05, meaning that no pleiotropy was found.

In addition, we reconfirmed the reliability of our results by examining forest plots, funnel plots, and scatter plots to detect potential outliers that may impact our MR estimates. In Supplementary material, the scatterplots, funnel plots, and forest plots are presented.

## 4 Discussion

In the present investigation, a two-sample MR approach was employed to explore the potential causal link between trace elements including Cu, Ir, Zn, Ca, VC, and VD, and the outcome of interest, GD. This analysis capitalized on publicly accessible summary statistics derived from GWAS and the UK Biobank dataset. Our MR analyses revealed that, excluding Cu, the remaining trace elements showed no direct causal relationship with GD.

In plasma, Cu binds to copper blue proteins and possesses the capacity to stimulate both intrinsic and adaptive immunity, which is crucial for the protective function of the immune system (33, 34). Analysis of data from the National Health and Nutrition Examination Survey (NHANES) reveals that Cu levels in men are associated with elevated levels of FT4 and Total Thyroxine 4 (TT4), whereas in women, Cu levels are positively correlated with elevated levels of Total Triiodothyronine (TT3) and TT4 (35). The extant literature postulates that Cu levels might influence Thyroid Stimulating Hormone (TSH) levels and the FT3: FT4 ratio by elevating FT4 levels (7). In addition, elevated Cu levels have been observed in association with hyperthyroidism, whereas patients suffering from Graves’ Ophthalmopathy (GO) exhibit a risk of experiencing decreased serum Cu levels (12). Several prior investigations have indicated that alterations in thyroid hormones have varying degrees of influence on the equilibrium of metal ions in erythrocytes and serum, particularly affecting Cu metabolism in cases of hyperthyroidism (36, 37). Animal studies conducted by Mittag et al. observed a significant elevation in Cu levels following thyroid hormone treatment (38). This phenomenon can be explained by the reduced expression of mRNA levels for competitive intracellular copper-binding proteins such as metallothioneins 1 and 2, as well as the increased synthesis and export of hepatic ceruloplasmin, the primary copper-carrying protein. Additionally, studies have found that copper exposure increases the levels of thyroid hormones T4 and T3 in fish eggs, suggesting that copper may disrupt the endocrine system (39). Research by Brookes et al. highlights the critical role of copper in enhancing the uptake of radioactive iodine in thyroid cancer cells, indicating that copper may promote thyroid hormone synthesis by increasing the activity of the sodium-iodide symporter (NIS) (40). Our research provides evidence of a potential causal relationship between genetically determined circulating Cu concentrations and GD.

Ir ions are abundantly present in numerous proteins, such as hemoglobin, myoglobin, and enzymes (6). Immune system performance and cognitive development are negatively impacted by its deficiency (25). Iron’s bioavailability regulates intricate metabolic pathways in inflammation and immune cell homeostasis (41). Plasma iron is responsible for regulating innate immunity through the modulation of the monocyte-to-neutrophil ratio and neutrophil activity (42). A meta-analysis revealed that patients with hyperthyroidism are at an increased risk of iron deficiency anemia compared to individuals with normal thyroid function (43). In West and North Africa, approximately 23–25% of school-age children exhibit both goiter and iron deficiency anemia (44). Iron supplementation also improves the effectiveness of iodized salt in children with goiter caused by iron deficiency (45). Furthermore, numerous studies have indicated that nutritional iron deficiency impacts thyroid metabolism, manifesting as decreased plasma TT3 and TT4 levels, reduced peripheral conversion of T4 to T3, and elevated TSH levels (25). These studies underscore the importance of Ir homeostasis in maintaining normal thyroid function. The mechanisms of iron uptake and storage in the thyroid are not yet fully understood. However, its role as a coenzyme for thyroid peroxidase (TPO) and its necessity for the efficiency of thyroid hormone synthesis has been well established (46). However, this study demonstrates that there is no causal relationship between genetically predicted iron cycling concentration and GD.

Zn impacts the production and regulation of thyroid hormone by modifying the activity of deiodinase, affecting the synthesis of Thyrotropin-Releasing Hormone (TRH) and TSH, and contributing to the formation of transcription factors essential for thyroid hormone synthesis. Kwon et al. found that zinc deficiency inhibits the synthesis and secretion of thyroglobulin by inducing endoplasmic reticulum stress, thereby affecting overall thyroid function (47). Simultaneously, thyroid hormones exert an influence on Zn metabolism, modulating Zn homeostasis *in vivo* through the regulation of zinc absorption and excretion processes. This intricate interplay is evident in the notable correlation between serum Zn concentrations and serum T3, T4, and TSH levels (16, 48). The extant research exploring the relationship between Zn and GD is limited, and the current investigation failed to establish a causal link between the circulating concentration of Zn and GD.

The dysregulation of Ca ion regulation in lymphocytes can result in perturbations in metabolism, proliferation, differentiation, antibody secretion, cytokine production, and cytotoxicity control, potentially leading to autoimmune and inflammatory diseases (49). Thyroid disorders play a pivotal role in mineral metabolism, specifically impacting the skeletal tissue’s volume of minerals. In hyperthyroidism, there is a significant increase in serum Ca content, increasing the chance of further fractures as well as osteoporosis. Calcium plays a crucial role in maintaining serum calcium homeostasis by influencing the secretion and gene expression of parathyroid hormone (PTH). Elevated serum calcium levels activate the calcium-sensing receptor (CaR), which in turn inhibits the stability of PTH mRNA and the secretion of PTH, thereby reducing PTH levels. CaR is a key regulator in this process, while vitamin D participates in this regulatory mechanism through various pathways (50). Previous studies have indicated an interaction between GD and *Ca.* However, in this study, we found that the MR-Egger and WME results demonstrated significant associations between Ca circulating concentrations and GD, whereas the IVW results did not show significant associations. This discrepancy is likely attributable to the potential bias in IVW results caused by horizontal pleiotropy. The MR-Egger method addresses this issue by allowing for a non-zero intercept, providing a more robust estimate even in the presence of pleiotropy. Despite the robustness and adaptability of the MR-Egger hypothesis, we opted for the IVW method due to its accuracy. Our findings suggest that there is no causal relationship between genetically predicted Ca cycle concentration and GD.

VC may contribute to the restoration of thyroid hormone synthesis functionality by safeguarding thyroid follicles from oxidative damage, subsequently facilitating thyroid hormone synthesis. In addition to its antioxidant properties, other non-antioxidant activities of vitamin C may similarly contribute to the restoration of thyroid function (51). A randomized controlled study revealed that patients with GD and active Graves’ Ophthalmopathy (GO) were categorized into hyperthyroid and euthyroid groups, based on their thyroid hormone levels. In comparison to healthy controls, individuals in the hyperthyroid group exhibited significantly decreased levels of vitamin C (52). Londzin-Olesik et al. observed that, in comparison to healthy controls, VC levels were reduced following the administration of systemic intravenous and oral methylprednisolone in patients with GD and GO (53). Previous studies have indicated that NADPH-cytochrome c reductase, supported by vitamin C, is crucial for the iodination of tyrosine in the presence of thyroid peroxidase, a key step in thyroid hormone synthesis (54). Various factors secreted by C cells, such as calcitonin, calcitonin gene-related peptide (CGRP), and gastrin-releasing peptide (GRP), can directly act on thyroid follicular cells in a paracrine manner, influencing the synthesis and secretion of thyroid hormones (such as T3 and T4). Vitamin C, through its antioxidant and immunomodulatory functions, reduces inflammatory responses and thus protects thyroid tissue from immune-mediated damage. It may play a supportive role in this paracrine regulation process (55). These studies suggest a potential bidirectional causal relationship between GD and VC. However, our analysis found no significant causal effect of VC’s cyclic concentrations on GD.

In recent decades, significant interest has arisen regarding the non-skeletal health benefits of VD, especially its implications in autoimmune diseases, metabolic syndrome, and cardiovascular disorders. Observational studies have demonstrated a significant decrease in serum 25(OH)D levels among AITD patients compared to control subjects, as well as lower levels in Thyroid Peroxidase Antibody (TPOAb) positive individuals compared to TPOAb negative individuals, independent of thyroid function status (56–58). Moreover, additional studies have linked low vitamin D levels to a higher risk of AITD and other autoimmune illnesses (59). Vitamin D has a relevant impact on PTH gene expression. Specifically, 1,25(OH)D regulates calcium by reducing PTH gene transcription, thereby indirectly influencing the synthesis of thyroid hormones (50). This study did not establish a clear causal effect between circulating VD concentrations and GD.

Previous investigations exploring the relationship between micronutrients and GD have generated inconsistent findings. To further delve into the potential causal association between them, we applied a two-sample MR approach in our research, marking the first application of this method. This MR method affords further proof that exposure and outcome are causally related while mitigating the limitations stemming from confounding factors that are prevalent in traditional observational studies. IVs in the study design comprised SNPs that exhibited strong correlation (*p* < 5 × 10^8^) and high intensity (F-statistic>10) with micronutrients, thereby substantially enhancing the comparability and credibility of the research. To mitigate population stratification, only European ancestry individuals were included. However, there are several limitations to the study. Firstly, Micronutrient instances, which have a relatively tiny sample size, hinder the ability to definitively exclude the existence of undetected weak associations, thereby compromising the comprehensiveness of the study. Secondly, the absence of detailed subgroup data impeded the conduct of more nuanced subgroup analyses, potentially overlooking specific associations within subgroups. Additionally, Graves’ disease patients may present with comorbidities, such as autoimmune disorders, which could potentially affect micronutrient metabolism and genetic associations. We propose that future research incorporate more detailed phenotypic data, including information on comorbidities, to enable a more precise and comprehensive exploration of the relationships between micronutrient levels, genetic factors, and GD. Meanwhile, although Mendelian randomization reduces confounding effects, the potential residual confounding from unmeasured environmental exposures in genome-wide association studies, the complexity of dietary influences, and data insufficiency may impact the results. Future studies incorporating more comprehensive dietary intake data are needed for further validation. Furthermore, the interplay between GD and trace elements may exhibit a reciprocal causal relationship, as opposed to solely discussing a unidirectional causal relationship in this study.

In considering the limitations of prior research, it is anticipated that future studies will advance in the following respects: Firstly, it is recommended to conduct a series of stratified experiments. In these experiments, relevant variables, including the dosage and duration of trace elements, must be rigorously controlled to enable a more precise examination of their impact on thyroid hormone and autoimmune antibody levels among GD patients. A deeper comprehension of the complex interactions between trace components and GD is made easier with the help of such a design. Secondly, to obtain a more holistic perspective on the impact of micronutrient levels on individuals with GD, it is recommended that research centers be established in several continents and nations, which would facilitate cross-national and cross-ethnic studies. Such international collaboration will not only facilitate a broader range of research samples, but will also elucidate the variations in the association between micronutrients and GD across diverse racial, geographic, and cultural settings, thereby providing a richer and more encompassing resource of data for MR studies. Finally, a bidirectional Mendelian randomization study could be considered to further elucidate the relationship between GD and trace elements. In the future, we will continue to explore and optimize research methods. At the same time, studies could also aim to validate the associations between these SNPs and micronutrient levels in the general population to ensure their consistency across different populations. Although the MR study used in this research can utilize genetic variations strongly correlated with exposure factors as instrumental variables to infer the causal effect between exposure factors and research outcomes, MR studies cannot replace randomized trials and should only serve as Supplementary material. It is recommended to conduct more traditional observational and experimental studies in the future to explain the relationship between the two, aiming to more comprehensively reveal the relationship between micronutrients and GD, and provide stronger scientific evidence for the prevention and treatment of related diseases.

## 5 Conclusion

This MR study demonstrated a causal relationship between Cu levels and an increased risk of GD, indicating that an increase in Cu levels could potentially elevate the risk of GD by 18.3% (*p* = 0.025). However, elements including Ir, Ca, Zn, VC, and VD failed to demonstrate significant associations with the risk of GD. Further research is necessary to elucidate the roles of micronutrients in GD and their underlying mechanisms.

## Data Availability

The original contributions presented in the study are included in the article/Supplementary material, further inquiries can be directed to the corresponding author.

## References

[1] Antonelli A Fallahi P Elia G Ragusa F Paparo SR Ruffilli I. Graves' disease: clinical manifestations, immune pathogenesis (cytokines and chemokines) and therapy. Best Pract Res Clin Endocrinol Metab. (2020) 34:101388. doi: 10.1016/j.beem.2020.101388 32059832

[2] Davies TF Andersen S Latif R Nagayama Y Barbesino G Brito M. Graves' disease. Nat Rev Dis Primers. (2020) 6:52. doi: 10.1038/s41572-020-0184-y 32616746

[3] McIver B Morris JC. The pathogenesis of Graves' disease. Endocrinol Metab Clin N Am. (1998) 27:73–89. doi: 10.1016/s0889-8529(05)70299-1 9534029

[4] Antonelli A Ferrari SM Ragusa F Elia G Paparo SR Ruffilli I. Graves' disease: epidemiology, genetic and environmental risk factors and viruses. Best Pract Res Clin Endocrinol Metab. (2020) 34:101387. doi: 10.1016/j.beem.2020.101387 32107168

[5] Duntas LH. Environmental factors and thyroid autoimmunity. Ann Endocrinol. (2011) 72:108–13. doi: 10.1016/j.ando.2011.03.019 21511233

[6] Kravchenko V Zakharchenko T. Thyroid hormones and minerals in Immunocorrection of disorders in autoimmune thyroid diseases. Front Endocrinol. (2023) 14:1225494. doi: 10.3389/fendo.2023.1225494, PMID: 37711890 PMC10499380

[7] Ye Y Li Y Ma Q Li Y Zeng H Luo Y. Association of Multiple Blood Metals with thyroid function in general adults: a Cross-sectional study. Front Endocrinol. (2023) 14:1134208. doi: 10.3389/fendo.2023.1134208, PMID: 37051196 PMC10083359

[8] Vrca VB Skreb F Cepelak I Romic Z Mayer L. Supplementation with antioxidants in the treatment of Graves' disease; the effect on glutathione peroxidase activity and concentration of Selenium. Clin Chim Acta. (2004) 341:55–63. doi: 10.1016/j.cccn.2003.10.028 14967159

[9] Xu MY Cao B Yin J Wang DF Chen KL Lu QB. Vitamin D and Graves' disease: a meta-analysis update. Nutrients. (2015) 7:3813–27. doi: 10.3390/nu7053813, PMID: 26007334 PMC4446781

[10] Nettore IC Albano L Ungaro P Colao A Macchia PE. Sunshine vitamin and thyroid. Rev Endocr Metab Disord. (2017) 18:347–54. doi: 10.1007/s11154-017-9406-3, PMID: 28092021 PMC5543192

[11] Rasic-Milutinovic Z Jovanovic D Bogdanovic G Trifunovic J Mutic J. Potential influence of Selenium, copper, zinc and cadmium on L-thyroxine substitution in patients with Hashimoto thyroiditis and hypothyroidism. Exp Clin Endocrinol Diabetes. (2017) 125:79–85. doi: 10.1055/s-0042-116070, PMID: 27793066

[12] Liu Y Liu S Mao J Piao S Qin J Peng S. Serum trace elements profile in Graves' disease patients with or without Orbitopathy in Northeast China. Biomed Res Int. (2018) 2018:1–8. doi: 10.1155/2018/3029379 PMC5818896 29546054

[13] Kim MJ Kim SC Chung S Kim S Yoon JW Park YJ. Exploring the role of copper and Selenium in the maintenance of Normal thyroid function among healthy Koreans. J Trace Elem Med Biol. (2020) 61:126558. doi: 10.1016/j.jtemb.2020.126558, PMID: 32480050

[14] Kandhro GA Kazi TG Afridi HI Kazi N Arain MB Sarfraz RA. Evaluation of Iron in serum and urine and their relation with thyroid function in female Goitrous patients. Biol Trace Elem Res. (2008) 125:203–12. doi: 10.1007/s12011-008-8174-z, PMID: 18568296

[15] Maywald M Wang F Rink L. The intracellular free zinc level is vital for Treg function and a feasible tool to discriminate between Treg and activated Th cells. Int J Mol Sci. (2018) 19:3575. doi: 10.3390/ijms19113575, PMID: 30428511 PMC6274670

[16] Ertek S Cicero AF Caglar O Erdogan G. Relationship between serum zinc levels, thyroid hormones and thyroid volume following successful iodine supplementation. Hormones (Athens). (2010) 9:263–8. doi: 10.14310/horm.2002.1276, PMID: 20688624

[17] Berridge MJ. The inositol trisphosphate/calcium signaling pathway in health and disease. Physiol Rev. (2016) 96:1261–96. doi: 10.1152/physrev.00006.2016, PMID: 27512009

[18] Me R Nema S Ee H. Assessment of serum level of calcium and phosphorus in Sudanese patients with hyperthyroidism. World J Pharm Pharm Sci. (2014) 3:20–7.

[19] Belosludtseva NV Talanov EY Venediktova NI Sharapov MG Mironova GD Belosludtsev KN. Structural and functional features of Ca(2+) transport systems in Liver Mitochondria of rats with experimental hyperthyroidism. Bull Exp Biol Med. (2020) 169:224–8. doi: 10.1007/s10517-020-04855-0 32654002

[20] Farasati Far B Behnoush AH Ghondaghsaz E Habibi MA Khalaji A. The interplay between vitamin C and thyroid. Endocrinol Diabetes Metab. (2023) 6:e432. doi: 10.1002/edm2.432, PMID: 37246589 PMC10335618

[21] Kim D. The role of vitamin D in thyroid diseases. Int J Mol Sci. (2017) 18:1949. doi: 10.3390/ijms18091949, PMID: 28895880 PMC5618598

[22] Altieri B Muscogiuri G Barrea L Mathieu C Vallone CV Mascitelli L. Does vitamin D play a role in autoimmune endocrine disorders? A proof of concept. Rev Endocr Metab Disord. (2017) 18:335–46. doi: 10.1007/s11154-016-9405-9, PMID: 28070798

[23] Cesareo R Attanasio R Caputo M Castello R Chiodini I Falchetti A. Italian Association of Clinical Endocrinologists (AME) and Italian chapter of the American Association of Clinical Endocrinologists (AACE) position statement: clinical management of vitamin D deficiency in adults. Nutrients. (2018) 10:546. doi: 10.3390/nu10050546, PMID: 29702603 PMC5986426

[24] Cooper DS. Subclinical thyroid disease: consensus or conundrum? Clin Endocrinol. (2004) 60:410–2. doi: 10.1111/j.1365-2265.2004.02031.x 15049953

[25] Zhou Q Xue S Zhang L Chen G. Trace elements and the thyroid. Front Endocrinol. (2022) 13:904889. doi: 10.3389/fendo.2022.904889, PMID: 36353227 PMC9637662

[26] Sekula P Del Greco MF Pattaro C Köttgen A. Mendelian randomization as an approach to assess causality using observational data. J Am Soc Nephrol. (2016) 27:3253–65. doi: 10.1681/asn.2016010098, PMID: 27486138 PMC5084898

[27] Bowden J Holmes MV. Meta-analysis and Mendelian randomization: a review. Res Synth Methods. (2019) 10:486–96. doi: 10.1002/jrsm.1346, PMID: 30861319 PMC6973275

[28] Burgess S Thompson SG. Avoiding Bias from weak instruments in Mendelian randomization studies. Int J Epidemiol. (2011) 40:755–64. doi: 10.1093/ije/dyr036 21414999

[29] Burgess S Thompson SG. Interpreting findings from Mendelian randomization using the Mr-egger method. Eur J Epidemiol. (2017) 32:377–89. Epub 20170519. doi: 10.1007/s10654-017-0255-x, PMID: 28527048 PMC5506233

[30] Auton A Brooks LD Durbin RM Garrison EP Kang HM Korbel JO. A global reference for human genetic variation. Nature. (2015) 526:68–74. doi: 10.1038/nature15393, PMID: 26432245 PMC4750478

[31] Lawlor DA Tilling K Davey SG. Triangulation in Aetiological epidemiology. Int J Epidemiol. (2016) 45:1866–86. doi: 10.1093/ije/dyw314, PMID: 28108528 PMC5841843

[32] Sanderson E Davey Smith G Windmeijer F Bowden J. An examination of multivariable Mendelian randomization in the single-sample and two-sample summary data settings. Int J Epidemiol. (2019) 48:713–27. doi: 10.1093/ije/dyy262, PMID: 30535378 PMC6734942

[33] Percival SS. Copper and immunity. Am J Clin Nutr. (1998) 67:1064s–8s. doi: 10.1093/ajcn/67.5.1064S 9587153

[34] Raha S Mallick R Basak S Duttaroy AK. Is copper beneficial for Covid-19 patients? Med Hypotheses. (2020) 142:109814. doi: 10.1016/j.mehy.2020.109814, PMID: 32388476 PMC7199671

[35] Jain RB. Thyroid function and serum copper, Selenium, and zinc in general U.S. population. Biol Trace Elem Res. (2014) 159:87–98. doi: 10.1007/s12011-014-9992-9 24789479

[36] Zhang F Liu N Wang X Zhu L Chai Z. Study of trace elements in blood of thyroid disorder subjects before and after 131i therapy. Biol Trace Elem Res. (2004) 97:125–34. doi: 10.1385/bter:97:2:125, PMID: 14985623

[37] Baltaci AK Mogulkoc R Belviranli M. L-thyroxine-induced hyperthyroidism affects elements and zinc in rats. Bratisl Lek Listy. (2013) 114:125–8. doi: 10.4149/bll_2013_027, PMID: 23406177

[38] Mittag J Behrends T Nordström K Anselmo J Vennström B Schomburg L. Serum copper as a novel biomarker for resistance to thyroid hormone. Biochem J. (2012) 443:103–9. doi: 10.1042/bj20111817, PMID: 22220593

[39] Suvi R Giovanna M Katja A. Experimental copper exposure, but not heat stress, leads to elevated Intraovarian thyroid hormone levels in three-Spined sticklebacks (*Gasterosteus Aculeatus*). Ecotoxicology. (2020) 29:1431–40. doi: 10.1007/s10646-020-02278-1, PMID: 32975733 PMC7581574

[40] Brookes K Zha L Kim J Kannappans V Wang W Sunassee K. A critical role for copper in enhanced Radioirdide uptake in thyroid Cancer cells. Cancer Res. (2023) 83:5053. doi: 10.1158/1538-7445.AM2023-5053

[41] Cronin SJF Woolf CJ Weiss G Penninger JM. The role of Iron regulation in Immunometabolism and immune-related disease. Front Mol Biosci. (2019) 6:116. doi: 10.3389/fmolb.2019.00116, PMID: 31824960 PMC6883604

[42] Frost JN Wideman SK Preston AE Teh MR Ai Z Wang L. Plasma Iron controls neutrophil production and function. Sci Adv. (2022) 8:eabq5384. doi: 10.1126/sciadv.abq5384, PMID: 36197985 PMC9534512

[43] Wopereis DM Du Puy RS van Heemst D Walsh JP Bremner A Bakker SJL. The relation between thyroid function and Anemia: a pooled analysis of individual participant data. J Clin Endocrinol Metab. (2018) 103:3658–67. doi: 10.1210/jc.2018-00481, PMID: 30113667 PMC6179176

[44] Zimmermann M Adou P Torresani T Zeder C Hurrell R. Persistence of goiter despite Oral iodine supplementation in Goitrous children with Iron deficiency Anemia in Côte D'ivoire. Am J Clin Nutr. (2000) 71:88–93. doi: 10.1093/ajcn/71.1.88, PMID: 10617951

[45] Hess SY Zimmermann MB Adou P Torresani T Hurrell RF. Treatment of Iron deficiency in Goitrous children improves the efficacy of iodized salt in Côte D'ivoire. Am J Clin Nutr. (2002) 75:743–8. doi: 10.1093/ajcn/75.4.743, PMID: 11916762

[46] Selenium KJ. Iodine and Iron-essential trace elements for thyroid hormone synthesis and metabolism. Int J Mol Sci. (2023) 24:3393. doi: 10.3390/ijms24043393, PMID: 36834802 PMC9967593

[47] Kwon K Lee E-R Kang K-H Hwang T-S Kim S-W Park H. Zinc depletion inhibits the synthesis and secretion of thyroglobulin by inducing endoplasmic reticulum stress in Pccl3 thyroid cells. Int J Biol Biomed Eng. (2022) 16:290–7. doi: 10.46300/91011.2022.16.36

[48] Severo JS Morais JBS de Freitas TEC Andrade ALP Feitosa MM Fontenelle LC. The role of zinc in thyroid hormones metabolism. Int J Vitam Nutr Res. (2019) 89:80–8. doi: 10.1024/0300-9831/a000262, PMID: 30982439

[49] Saini N Lakshminarayanan S Kundu P Sarin A. Notch1 modulation of cellular calcium regulates mitochondrial metabolism and anti-apoptotic activity in T-regulatory cells. Front Immunol. (2022) 13:832159. doi: 10.3389/fimmu.2022.832159 35222416 PMC8866856

[50] Naveh-Many T Nechama M. Molecular mechanisms of parathyroid hormone synthesis. In: Licata AA, Lerma EV, editors. Diseases of the Parathyroid Glands. New York, NY: Springer New York (2012) p. 1–12.

[51] Ambali SF Orieji C Abubakar WO Shittu M Kawu MU. Ameliorative effect of vitamin C on alterations in thyroid hormones concentrations induced by subchronic Coadministration of Chlorpyrifos and Lead in Wistar rats. J Thyroid Res. (2011) 2011:214924:1–6. doi: 10.4061/2011/214924, PMID: 21687644 PMC3112502

[52] Londzin-Olesik M Kos-Kudła B Nowak A Wielkoszyński T Nowak M. The effect of thyroid hormone status on selected antioxidant parameters in patients with Graves' disease and active thyroid-associated Orbitopathy. Endokrynol Pol. (2020) 71:418–24. doi: 10.5603/EP.a2020.0049, PMID: 32797475

[53] Londzin-Olesik M Kos-Kudla B Karpe J Nowak A Nowak M. The effect of immunosuppression on selected antioxidant parameters in patients with Graves' disease with active thyroid-associated Orbitopathy. Exp Clin Endocrinol Diabetes. (2021) 129:762–9. doi: 10.1055/a-1274-0998, PMID: 33157557

[54] Yamamoto K DeGroot LJ. Participation of Nadph-cytochrome C reductase in thyroid hormone biosynthesis. Endocrinology. (1975) 96:1022–9. doi: 10.1210/endo-96-4-1022, PMID: 235416

[55] Fernández-Santos J Morillo-Bernal J García-Marín R Utrilla JC Martín-Lacave I. Paracrine regulation of thyroid-hormone synthesis by C cells. In: Agrawal NK, editors. Thyroid Hormone. IntechOpen (2012) p. 51–84.

[56] Kivity S Agmon-Levin N Zisappl M Shapira Y Nagy EV Dankó K. Vitamin D and autoimmune thyroid diseases. Cell Mol Immunol. (2011) 8:243–7. doi: 10.1038/cmi.2010.73, PMID: 21278761 PMC4012880

[57] Bozkurt NC Karbek B Ucan B Sahin M Cakal E Ozbek M. The association between severity of vitamin D deficiency and Hashimoto's thyroiditis. Endocr Pract. (2013) 19:479–84. doi: 10.4158/ep12376.Or, PMID: 23337162

[58] Shin DY Kim KJ Kim D Hwang S Lee EJ. Low serum vitamin D is associated with anti-thyroid peroxidase antibody in autoimmune thyroiditis. Yonsei Med J. (2014) 55:476–81. doi: 10.3349/ymj.2014.55.2.476, PMID: 24532520 PMC3936621

[59] Bouillon R Marcocci C Carmeliet G Bikle D White JH Dawson-Hughes B. Skeletal and Extraskeletal actions of vitamin D: current evidence and outstanding questions. Endocr Rev. (2019) 40:1109–51. doi: 10.1210/er.2018-00126, PMID: 30321335 PMC6626501

