# Supplementary material for: Effect of micronutrients on the risk of Graves’ disease: a Mendelian randomization study
Source: Front Nutr. 2024 Dec 9;11:1432420. doi: 10.3389/fnut.2024.1432420 (PMC11663635; doi:10.3389/fnut.2024.1432420)
Supplement: Supplementary file 1 [file Data_Sheet_1.docx]

***Supplementary Material***

1. **Supplementary Figures and Tables**
   1. **Supplementary Figures**

**
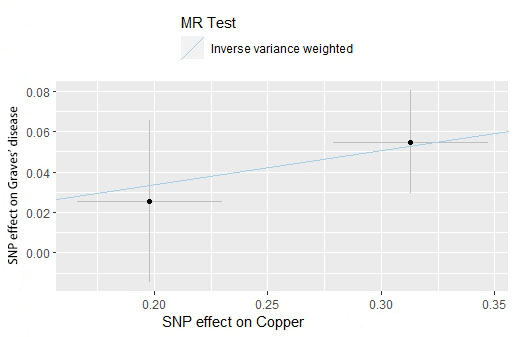
**

**Figure S1： Scatter plot of SNPs associated with Cu and their risk of Graves' disease.**

**
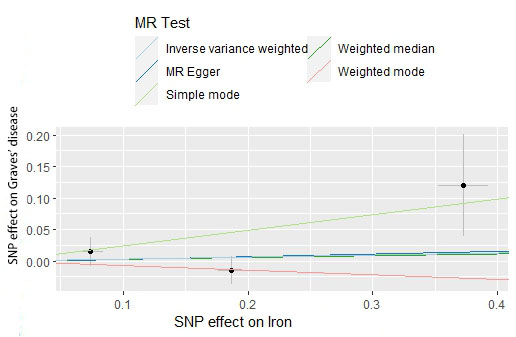
**

**Figure S2： Scatter plot of SNPs associated with Ir and their risk of Graves' disease.**

**
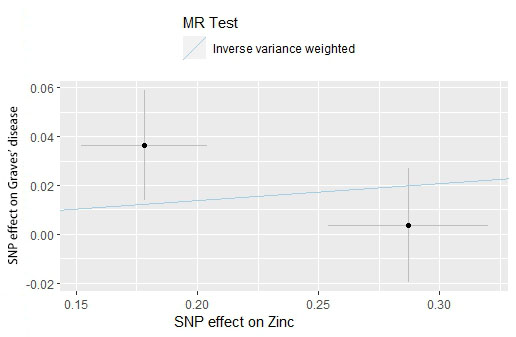
**

**Figure S3： Scatter plot of SNPs associated with Zn and their risk of Graves' disease.**

**
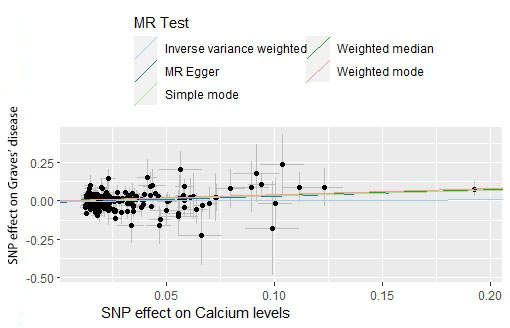
**

**Figure S4： Scatter plot of SNPs associated with Ca and their risk of Graves' disease.**

**
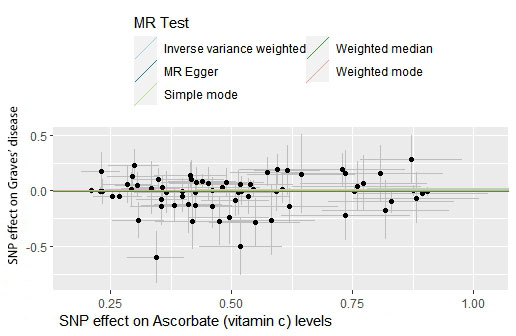
**

**Figure S5： Scatter plot of SNPs associated with VC and their risk of Graves' disease.**

**
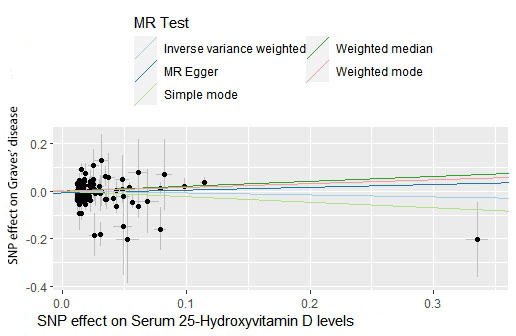
**

**Figure S6： Scatter plot of SNPs associated with VD and their risk of Graves' disease.**

**
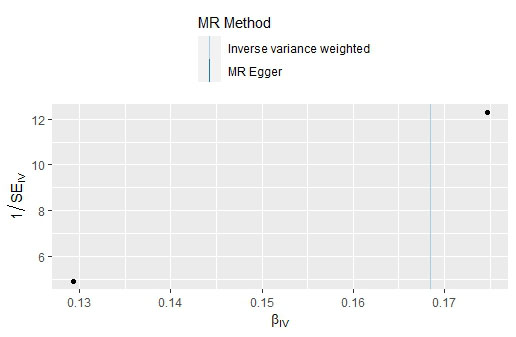
**

**Figure S7：Funnel plot of SNPs associated with Cu and their risk of Graves' disease.**

**
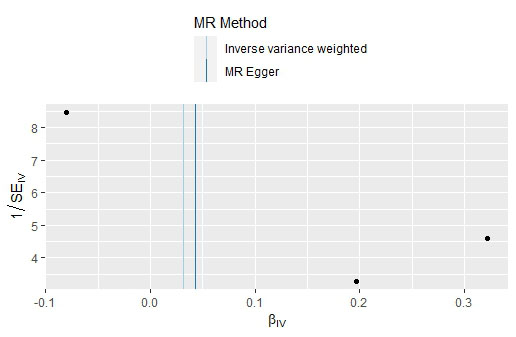
**

**Figure S8：Funnel plot of SNPs associated with Ir and their risk of Graves' disease.**

**
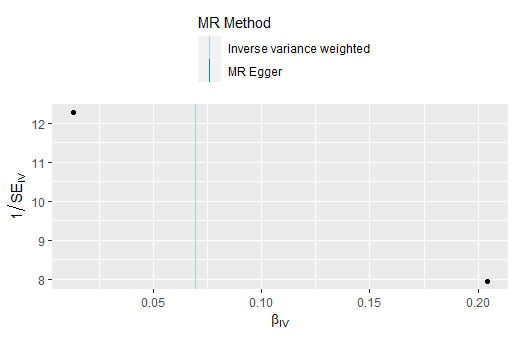
**

**Figure S9：Funnel plot of SNPs associated with Zn and their risk of Graves' disease.**

**
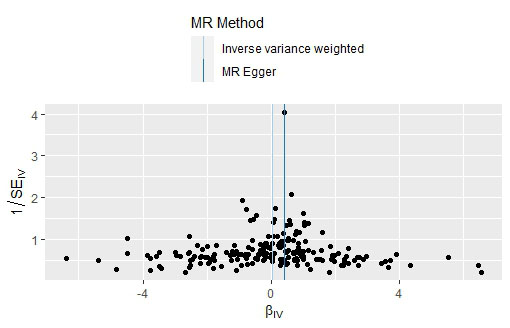
**

**Figure S10：Funnel plot of SNPs associated with Ca and their risk of Graves' disease.**

**
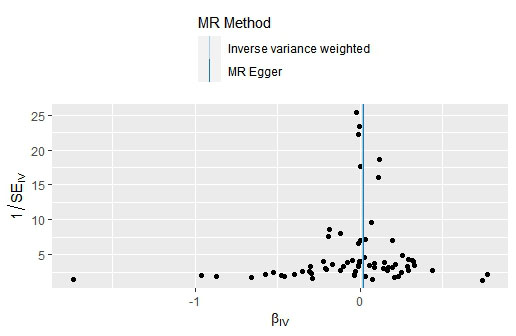
**

**Figure S11：Funnel plot of SNPs associated with VC and their risk of Graves' disease.**

**
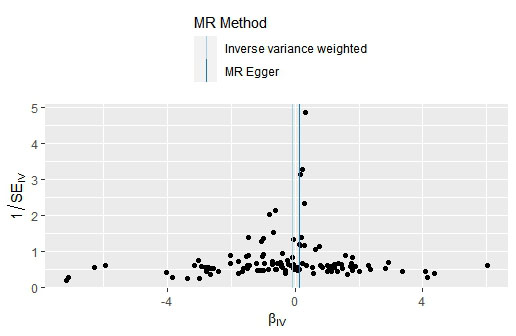
**

**Figure S12：Funnel plot of SNPs associated with VD and their risk of Graves' disease.**

**
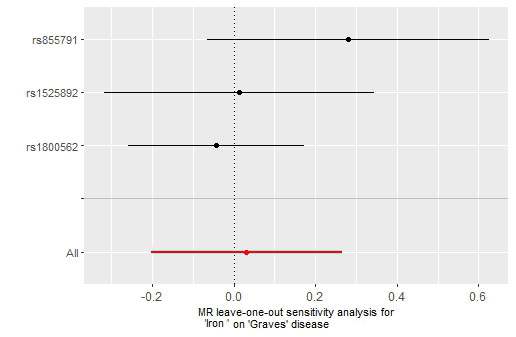
**

**Figure S13: Forest plot of SNPs associated with Ir and their risk of Graves' disease.**

**
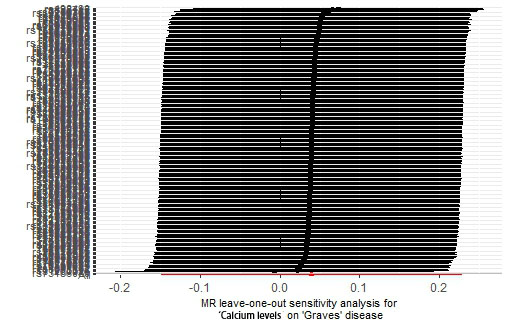
**

**Figure S14: Forest plot of SNPs associated with Ca and their risk of Graves' disease.**

**
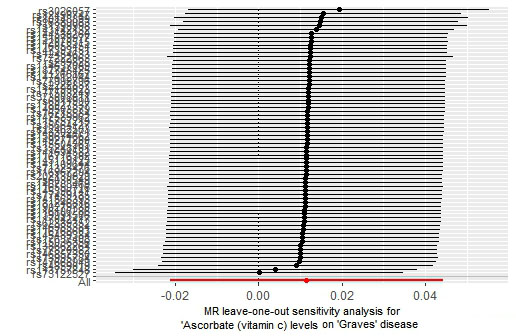
**

**Figure S15: Forest plot of SNPs associated with VC and their risk of Graves' disease.**

**
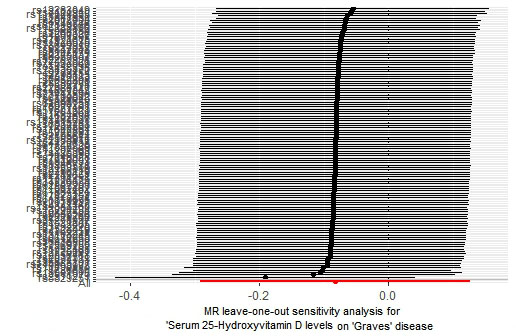
**

**Figure S16: Forest plot of SNPs associated with VD and their risk of Graves' disease.**

**
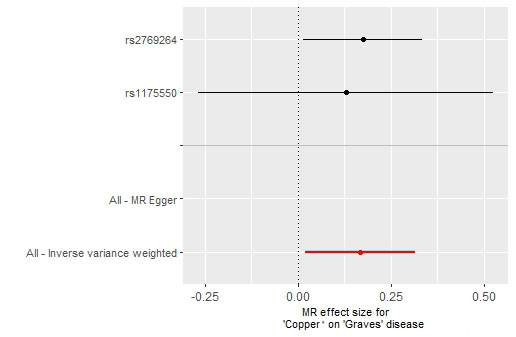
**

**Figure S17: Forest plot of SNPs associated with Cu and their risk of Graves' disease.**

**
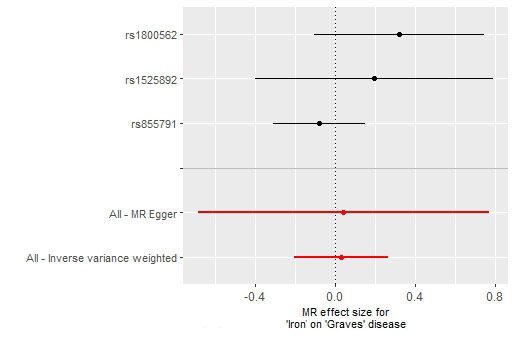
**

**Figure S18: Forest plot of SNPs associated with Ir and their risk of Graves' disease.**

**
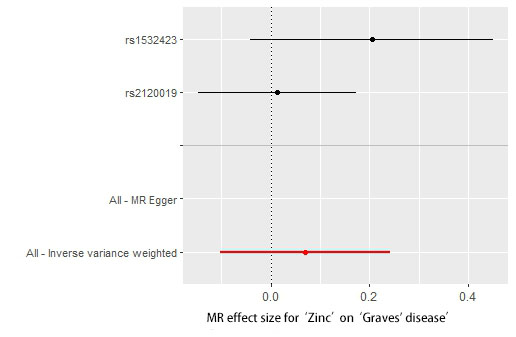
**

**Figure S19: Forest plot of SNPs associated with Zn and their risk of Graves' disease.**

**
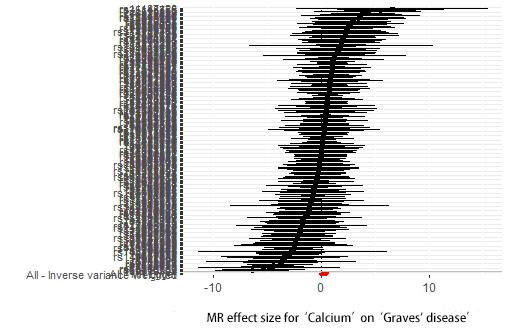
**

**Figure S20: Forest plot of SNPs associated with Ca and their risk of Graves' disease.**

**
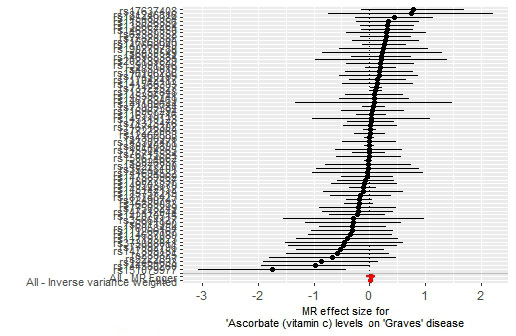
**

**Figure S21: Forest plot of SNPs associated with VC and their risk of Graves' disease.**

**
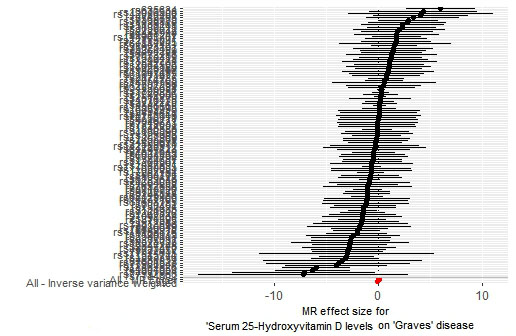
**

**Figure S22: Forest plot of SNPs associated with VD and their risk of Graves' disease.**

| **SNP** | **chr** | **pos** | **beta** | **se** | **effect_allele** | **Pval** | **F** |
| --- | --- | --- | --- | --- | --- | --- | --- |
| rs1175550 | 1 | 3691528 | 0.198 | 0.032 | G | 5.03E-10 | 38.28516 |
| rs2769264 | 1 | 1.51E+08 | 0.313 | 0.034 | G | 2.63E-20 | 84.74827 |

- 1. **Supplementary Figures**

**Supplementary Table 1. 2 SNPs associated with Copper.**

**Supplementary Table 2. 3 SNPs associated with Iron.**

| **SNP** | **chr** | **Pos** | **beta** | **se** | **effect_allele** | **pval** | **F** |
| --- | --- | --- | --- | --- | --- | --- | --- |
| rs1525892 | 3 | 1.33E+08 | 0.0736 | 0.0104 | A | 1.65E-12 | 50.08284 |
| rs1800562 | 6 | 26093141 | 0.3724 | 0.02 | A | 3.96E-77 | 346.7044 |
| rs855791 | 22 | 37462936 | 0.1868 | 0.0101 | G | 4.31E-77 | 342.0669 |

**Supplementary Table 3. 2 SNPs associated with Zinc.**

| **SNP** | **chr** | **pos** | **beta** | **se** | **effect_allele** | **pval** | **F** |
| --- | --- | --- | --- | --- | --- | --- | --- |
| rs1532423 | 8 | 86268313 | -0.178 | 0.026 | G | 6.40E-12 | 46.86982 |
| rs2120019 | 15 | 75334184 | -0.287 | 0.033 | C | 1.55E-18 | 75.63728 |

**Supplementary Table 4. 212 SNPs associated with Calcium.**

| **SNP** | **chr** | **pos** | **beta** | **se** | **effect_allele** | **pval** | **F** |
| --- | --- | --- | --- | --- | --- | --- | --- |
| rs75895430 | 1 | 68268784 | 0.0796 | 0.0071 | G | 5.31E-29 | 125.6925 |
| rs71658797 | 1 | 77967507 | 0.0211 | 0.0038 | A | 2.36E-08 | 30.83172 |
| rs9428344 | 1 | 1.17E+08 | 0.0124 | 0.0022 | T | 2.22E-08 | 31.7686 |
| rs12135382 | 1 | 1098421 | 0.0222 | 0.0026 | T | 1.29E-17 | 72.90533 |
| rs12132412 | 1 | 21820042 | 0.0276 | 0.0024 | G | 8.06E-31 | 132.25 |
| rs12411216 | 1 | 1.55E+08 | 0.0254 | 0.0023 | A | 3.87E-28 | 121.9584 |
| rs10917386 | 1 | 23799001 | 0.0192 | 0.0023 | T | 2.36E-16 | 69.6862 |
| rs11204766 | 1 | 1.51E+08 | 0.0411 | 0.0045 | C | 1.30E-19 | 83.41778 |
| rs7546838 | 1 | 1.57E+08 | 0.017 | 0.0024 | G | 1.67E-12 | 50.17361 |
| rs11588907 | 1 | 1.79E+08 | -0.0159 | 0.0026 | T | 1.19E-09 | 37.39793 |
| rs1434282 | 1 | 1.99E+08 | 0.0196 | 0.0025 | T | 8.03E-15 | 61.4656 |
| rs116631899 | 1 | 52787887 | 0.0732 | 0.0088 | C | 8.00E-17 | 69.19215 |
| rs697852 | 1 | 2.27E+08 | -0.0207 | 0.0032 | A | 1.65E-10 | 41.84473 |
| rs924204 | 1 | 16513926 | 0.0195 | 0.0023 | G | 7.10E-17 | 71.88091 |
| rs841572 | 1 | 43436051 | 0.0297 | 0.0022 | A | 1.52E-40 | 182.25 |
| rs1497826 | 1 | 2.17E+08 | 0.0229 | 0.0024 | G | 3.66E-21 | 91.0434 |
| rs10863512 | 1 | 2.2E+08 | -0.0199 | 0.0023 | T | 2.72E-18 | 74.86011 |
| rs116769926 | 2 | 9007708 | 0.0726 | 0.0082 | A | 6.58E-19 | 78.38727 |
| rs12998379 | 2 | 10163922 | -0.0219 | 0.0029 | A | 7.17E-14 | 57.02854 |
| rs76170039 | 2 | 54846795 | 0.0203 | 0.0031 | TAA | 5.78E-11 | 42.88137 |
| rs35751693 | 2 | 98242555 | 0.0433 | 0.0066 | T | 5.15E-11 | 43.04155 |
| rs778368 | 2 | 2.34E+08 | -0.0128 | 0.0023 | C | 1.70E-08 | 30.97164 |
| rs838717 | 2 | 2.34E+08 | -0.0431 | 0.0022 | A | 4.42E-84 | 383.8037 |
| rs74847504 | 2 | 69639612 | 0.016 | 0.0022 | G | 5.38E-13 | 52.89256 |
| rs1667305 | 2 | 2.32E+08 | 0.0125 | 0.0023 | C | 3.06E-08 | 29.53686 |
| rs10182990 | 2 | 2.42E+08 | -0.0205 | 0.0026 | G | 3.36E-15 | 62.16716 |
| rs35095338 | 2 | 1.91E+08 | 0.0147 | 0.0022 | T | 3.29E-11 | 44.64669 |
| rs7559013 | 2 | 2.14E+08 | 0.0253 | 0.0037 | C | 7.55E-12 | 46.75603 |
| rs2971855 | 2 | 2.34E+08 | 0.0172 | 0.0025 | A | 8.01E-12 | 47.3344 |
| rs1260326 | 2 | 27730940 | -0.0464 | 0.0022 | C | 1.25E-95 | 444.8264 |
| rs1374161 | 2 | 1.14E+08 | -0.0206 | 0.0023 | A | 8.89E-20 | 80.21928 |
| rs3748861 | 2 | 1.33E+08 | -0.0169 | 0.0028 | A | 2.44E-09 | 36.42985 |
| rs13389219 | 2 | 1.66E+08 | -0.0162 | 0.0024 | T | 2.68E-11 | 45.5625 |
| rs146582485 | 2 | 10122470 | -0.021 | 0.0031 | CGCAGTGGTTT | 1.96E-11 | 45.8897 |
| rs7587636 | 2 | 25518862 | -0.015 | 0.0022 | A | 2.47E-11 | 46.4876 |
| rs4430924 | 2 | 61703856 | -0.028 | 0.0025 | G | 2.90E-28 | 125.44 |
| rs72941253 | 2 | 97392707 | -0.0382 | 0.0025 | G | 3.12E-51 | 233.4784 |
| rs6542581 | 2 | 1.21E+08 | -0.022 | 0.0023 | G | 7.45E-21 | 91.49338 |
| rs7592216 | 2 | 1.83E+08 | -0.0206 | 0.0036 | T | 8.65E-09 | 32.74383 |
| rs715 | 2 | 2.12E+08 | -0.0138 | 0.0025 | C | 3.07E-08 | 30.4704 |
| rs147836956 | 3 | 1.22E+08 | -0.0626 | 0.0085 | ACT | 1.48E-13 | 54.23889 |
| rs4685773 | 3 | 4632637 | 0.0293 | 0.0041 | T | 8.65E-13 | 51.0702 |
| rs1899951 | 3 | 12394840 | -0.0346 | 0.0036 | T | 2.58E-21 | 92.37346 |
| rs10513810 | 3 | 1.87E+08 | -0.0268 | 0.0039 | G | 4.19E-12 | 47.22156 |
| rs648514 | 3 | 52467263 | -0.0144 | 0.0022 | A | 1.03E-10 | 42.84298 |
| rs1354034 | 3 | 56849749 | -0.016 | 0.0022 | C | 7.29E-13 | 52.89256 |
| rs141079827 | 3 | 1.21E+08 | -0.0555 | 0.0091 | A | 1.12E-09 | 37.19659 |
| rs34395935 | 3 | 1.22E+08 | 0.0576 | 0.0033 | C | 9.92E-67 | 304.6612 |
| rs73186030 | 3 | 1.22E+08 | 0.1927 | 0.0036 | T | ####### | 2865.223 |
| rs76249487 | 3 | 1.22E+08 | -0.0395 | 0.0048 | A | 1.17E-16 | 67.71918 |
| rs140559425 | 3 | 1.57E+08 | -0.0149 | 0.0022 | AAAT | 3.35E-11 | 45.86983 |
| rs10049088 | 3 | 1.57E+08 | 0.0139 | 0.0022 | T | 6.86E-10 | 39.91942 |
| rs138789759 | 3 | 1.07E+08 | 0.0469 | 0.0047 | A | 3.47E-23 | 99.57492 |
| rs73186098 | 3 | 1.22E+08 | -0.0914 | 0.0106 | C | 8.41E-18 | 74.34995 |
| rs3905668 | 3 | 1.36E+08 | 0.0258 | 0.0027 | G | 6.86E-21 | 91.30864 |
| rs9811323 | 3 | 1.78E+08 | 0.0148 | 0.0026 | C | 1.87E-08 | 32.40237 |
| rs13073106 | 3 | 1.86E+08 | 0.0341 | 0.0023 | T | 2.41E-48 | 219.8129 |
| rs6841258 | 4 | 40565426 | -0.0361 | 0.0029 | T | 1.51E-34 | 154.9596 |
| rs183515408 | 4 | 1.06E+08 | 0.0226 | 0.0037 | T | 1.17E-09 | 37.30898 |
| rs77849807 | 4 | 26152727 | 0.0566 | 0.01 | G | 1.36E-08 | 32.0356 |
| rs72660383 | 4 | 75949596 | -0.0261 | 0.0046 | C | 1.58E-08 | 32.19329 |
| rs11730491 | 4 | 26174563 | 0.0169 | 0.0029 | T | 4.34E-09 | 33.96076 |
| rs7655631 | 4 | 26869338 | -0.0148 | 0.0024 | C | 7.12E-10 | 38.02778 |
| rs7688574 | 4 | 38533499 | 0.0139 | 0.0025 | T | 3.30E-08 | 30.9136 |
| rs62309863 | 4 | 1.15E+08 | -0.0159 | 0.0022 | T | 1.11E-12 | 52.23347 |
| rs13108218 | 4 | 3443931 | -0.0393 | 0.0023 | G | 2.22E-67 | 291.9641 |
| rs13107325 | 4 | 1.03E+08 | -0.064 | 0.0047 | T | 5.42E-42 | 185.4233 |
| rs4320103 | 4 | 1.71E+08 | 0.0348 | 0.0057 | G | 8.52E-10 | 37.27424 |
| rs62362193 | 5 | 72345509 | 0.0163 | 0.0023 | A | 2.84E-12 | 50.22495 |
| rs4976647 | 5 | 1.77E+08 | 0.02 | 0.0023 | C | 7.35E-18 | 75.61437 |
| rs35096828 | 5 | 1.34E+08 | -0.0233 | 0.0026 | C | 1.16E-18 | 80.30917 |
| rs11743466 | 5 | 1.74E+08 | 0.0145 | 0.0023 | G | 3.88E-10 | 39.7448 |
| rs10942734 | 5 | 74595194 | 0.015 | 0.0022 | C | 1.44E-11 | 46.4876 |
| rs657075 | 5 | 1.31E+08 | -0.0177 | 0.0032 | A | 3.91E-08 | 30.59473 |
| rs9390702 | 6 | 1.01E+08 | 0.0129 | 0.0022 | T | 7.65E-09 | 34.38223 |
| rs9401792 | 6 | 1.25E+08 | -0.0216 | 0.0023 | G | 4.57E-20 | 88.1966 |
| rs945890 | 6 | 1.3E+08 | -0.0149 | 0.0024 | T | 1.06E-09 | 38.5434 |
| rs1763519 | 6 | 1.35E+08 | -0.0295 | 0.0023 | C | 1.54E-36 | 164.5085 |
| rs490275 | 6 | 1.61E+08 | -0.0134 | 0.0022 | T | 2.46E-09 | 37.09917 |
| rs6597256 | 6 | 7208007 | -0.0127 | 0.0023 | A | 2.06E-08 | 30.4896 |
| rs9379881 | 6 | 10877867 | -0.0136 | 0.0022 | C | 9.96E-10 | 38.21488 |
| rs915896 | 6 | 32187721 | -0.0168 | 0.0023 | T | 7.15E-13 | 53.3535 |
| rs2327774 | 6 | 1.37E+08 | -0.0199 | 0.0023 | C | 1.15E-17 | 74.86011 |
| rs1933737 | 6 | 1.16E+08 | -0.0147 | 0.0025 | C | 8.21E-09 | 34.5744 |
| rs1187115 | 6 | 34172055 | -0.0425 | 0.0033 | A | 6.14E-38 | 165.8632 |
| rs9356996 | 6 | 26008487 | -0.0166 | 0.0024 | A | 5.27E-12 | 47.84028 |
| rs7769064 | 6 | 74495849 | -0.0393 | 0.0023 | G | 5.08E-63 | 291.9641 |
| rs212837 | 7 | 26695215 | 0.0127 | 0.0023 | C | 2.89E-08 | 30.4896 |
| rs17164683 | 7 | 92286980 | -0.0216 | 0.0028 | T | 7.32E-15 | 59.5102 |
| rs11772303 | 7 | 1.27E+08 | 0.013 | 0.0024 | T | 3.71E-08 | 29.34028 |
| rs4718271 | 7 | 65212402 | 0.0307 | 0.0025 | C | 6.87E-35 | 150.7984 |
| rs7786368 | 7 | 77500734 | -0.0262 | 0.0023 | C | 4.94E-30 | 129.7618 |
| rs62483619 | 7 | 1.07E+08 | -0.0215 | 0.0025 | T | 2.28E-17 | 73.96 |
| rs3857708 | 7 | 16140077 | 0.0192 | 0.0025 | A | 3.57E-14 | 58.9824 |
| rs114949263 | 7 | 1.5E+08 | 0.034 | 0.0039 | C | 7.86E-18 | 76.00263 |
| rs41393948 | 8 | 8296230 | -0.018 | 0.0031 | T | 1.10E-08 | 33.71488 |
| rs4841132 | 8 | 9183596 | 0.0583 | 0.0042 | G | 4.68E-43 | 192.6808 |
| rs4647903 | 8 | 38272582 | 0.019 | 0.0025 | A | 6.67E-14 | 57.76 |
| rs2309233 | 8 | 21945518 | 0.0213 | 0.0028 | C | 5.91E-14 | 57.86862 |
| rs36104352 | 8 | 23377604 | 0.0239 | 0.0034 | C | 1.89E-12 | 49.41263 |
| rs2343592 | 8 | 1.07E+08 | -0.0216 | 0.0024 | G | 7.71E-19 | 81 |
| rs12334564 | 8 | 1.25E+08 | -0.0136 | 0.0024 | A | 1.29E-08 | 32.11111 |
| rs2016749 | 8 | 1.44E+08 | -0.0143 | 0.0025 | A | 1.78E-08 | 32.7184 |
| rs7003580 | 8 | 1.45E+08 | 0.015 | 0.0023 | T | 1.19E-10 | 42.53308 |
| rs16930077 | 8 | 63920229 | -0.0169 | 0.0026 | A | 6.57E-11 | 42.25 |
| rs7839633 | 8 | 98812248 | -0.0123 | 0.0022 | G | 2.75E-08 | 31.25826 |
| rs3133548 | 8 | 1.02E+08 | 0.0163 | 0.0029 | T | 2.33E-08 | 31.59215 |
| rs12378991 | 9 | 77472066 | -0.0382 | 0.0046 | A | 9.51E-17 | 68.96219 |
| rs12337706 | 9 | 97523154 | -0.0514 | 0.0042 | G | 1.88E-34 | 149.771 |
| rs550057 | 9 | 1.36E+08 | -0.0142 | 0.0025 | T | 1.58E-08 | 32.2624 |
| rs296849 | 9 | 4780007 | 0.0123 | 0.0022 | A | 3.27E-08 | 31.25826 |
| rs7856502 | 9 | 904078 | -0.0196 | 0.0026 | T | 5.22E-14 | 56.8284 |
| rs883951 | 9 | 71514547 | 0.0233 | 0.0026 | G | 1.02E-18 | 80.30917 |
| rs7864156 | 9 | 96905219 | 0.0191 | 0.0025 | G | 6.61E-14 | 58.3696 |
| rs4978466 | 9 | 1.15E+08 | -0.0143 | 0.0026 | A | 3.02E-08 | 30.25 |
| rs4744854 | 9 | 80498559 | -0.0283 | 0.0023 | C | 2.48E-33 | 151.397 |
| rs518636 | 9 | 1.16E+08 | 0.0138 | 0.0025 | G | 3.24E-08 | 30.4704 |
| rs10739679 | 9 | 1.29E+08 | 0.0325 | 0.0023 | G | 3.33E-45 | 199.6692 |
| rs498490 | 10 | 8118677 | -0.0261 | 0.0028 | T | 3.27E-20 | 86.88903 |
| rs112371897 | 10 | 9321880 | 0.0698 | 0.0042 | T | 1.06E-60 | 276.1927 |
| rs12416595 | 10 | 22409964 | 0.0152 | 0.0027 | G | 2.25E-08 | 31.69273 |
| rs17774672 | 10 | 50507709 | -0.0288 | 0.0032 | A | 1.16E-19 | 81 |
| rs9415676 | 10 | 65010626 | 0.0147 | 0.0023 | G | 2.59E-10 | 40.84877 |
| rs5786388 | 10 | 80999929 | 0.0188 | 0.0022 | CA | 3.93E-17 | 73.02479 |
| rs1061134 | 10 | 1E+08 | -0.0216 | 0.0038 | A | 8.71E-09 | 32.31025 |
| rs7086226 | 10 | 22459979 | 0.0191 | 0.0026 | G | 5.31E-13 | 53.96598 |
| rs4082330 | 10 | 65497266 | 0.0208 | 0.0032 | T | 1.12E-10 | 42.25 |
| rs11187128 | 10 | 94429708 | -0.0148 | 0.0025 | T | 4.78E-09 | 35.0464 |
| rs2419886 | 10 | 1.16E+08 | -0.0192 | 0.0026 | T | 2.99E-13 | 54.53254 |
| rs2762630 | 10 | 9265199 | 0.0317 | 0.0027 | G | 2.83E-31 | 137.845 |
| rs4935009 | 10 | 52829393 | -0.0193 | 0.003 | C | 2.33E-10 | 41.38778 |
| rs11187838 | 10 | 96038686 | -0.018 | 0.0022 | A | 4.97E-16 | 66.94215 |
| rs4938642 | 11 | 1.19E+08 | 0.0287 | 0.0037 | C | 1.81E-14 | 60.16728 |
| rs73632745 | 11 | 1.26E+08 | -0.0611 | 0.0048 | T | 9.31E-38 | 162.0317 |
| rs2004315 | 11 | 13508384 | 0.0315 | 0.0023 | T | 1.93E-42 | 187.5709 |
| rs12793417 | 11 | 34622050 | 0.0172 | 0.0026 | T | 8.03E-11 | 43.76331 |
| rs3841466 | 11 | 77925543 | -0.0158 | 0.0027 | TG | 9.86E-09 | 34.24417 |
| rs7108820 | 11 | 1.02E+08 | 0.0143 | 0.0022 | C | 1.98E-10 | 42.25 |
| rs2583435 | 11 | 2958818 | -0.0233 | 0.0024 | C | 2.96E-22 | 94.25174 |
| rs144562710 | 11 | 47933609 | 0.02 | 0.0026 | AAT | 1.37E-14 | 59.1716 |
| rs4517550 | 11 | 71521071 | 0.0146 | 0.0023 | C | 3.91E-10 | 40.2949 |
| rs302655 | 11 | 87891387 | -0.0186 | 0.0023 | T | 1.56E-16 | 65.39887 |
| rs11228382 | 11 | 68617316 | -0.0182 | 0.0022 | T | 5.67E-16 | 68.43802 |
| rs1182922 | 11 | 1.19E+08 | -0.0134 | 0.0023 | A | 5.13E-09 | 33.94329 |
| rs949300 | 11 | 1.23E+08 | 0.0146 | 0.0023 | A | 1.20E-10 | 40.2949 |
| rs775249 | 12 | 57977003 | -0.0155 | 0.0025 | T | 4.00E-10 | 38.44 |
| rs3026445 | 12 | 1.11E+08 | -0.0172 | 0.0025 | C | 1.45E-11 | 47.3344 |
| rs17884869 | 12 | 1.24E+08 | -0.1113 | 0.0079 | A | 1.05E-44 | 198.4889 |
| rs73202933 | 12 | 90213083 | 0.0235 | 0.0033 | C | 9.88E-13 | 50.71166 |
| rs117213754 | 12 | 4006794 | 0.1036 | 0.0102 | A | 3.05E-24 | 103.1619 |
| rs117080167 | 12 | 12205320 | -0.0318 | 0.0045 | T | 2.35E-12 | 49.93778 |
| rs7964801 | 12 | 49097212 | -0.0271 | 0.0026 | T | 1.43E-24 | 108.6405 |
| rs6580981 | 12 | 54723028 | -0.0152 | 0.0023 | A | 1.29E-11 | 43.67486 |
| rs7323058 | 13 | 42548503 | -0.0348 | 0.0034 | T | 7.06E-24 | 104.7612 |
| rs9524868 | 13 | 95909661 | -0.0135 | 0.0024 | T | 1.15E-08 | 31.64063 |
| rs2249825 | 13 | 31037903 | -0.0165 | 0.0026 | C | 2.55E-10 | 40.27367 |
| rs1577452 | 13 | 1.1E+08 | -0.0212 | 0.0025 | G | 4.88E-17 | 71.9104 |
| rs1023229 | 13 | 20270925 | -0.02 | 0.0034 | A | 7.21E-09 | 34.60208 |
| rs35852840 | 14 | 64595763 | 0.029 | 0.0053 | A | 4.30E-08 | 29.93948 |
| rs17580 | 14 | 94847262 | 0.0466 | 0.0058 | A | 5.51E-16 | 64.55291 |
| rs58087925 | 14 | 1.06E+08 | -0.0215 | 0.0029 | T | 2.83E-13 | 54.96433 |
| rs28693943 | 14 | 60640916 | -0.0192 | 0.0029 | T | 7.66E-11 | 43.83353 |
| rs28929474 | 14 | 94844947 | 0.1231 | 0.0088 | T | 2.20E-44 | 195.6819 |
| rs11621792 | 14 | 24871926 | 0.0156 | 0.0024 | T | 1.54E-10 | 42.25 |
| rs7144433 | 14 | 90850229 | -0.0252 | 0.0033 | T | 4.76E-14 | 58.31405 |
| rs11629876 | 15 | 96666402 | -0.0157 | 0.0024 | T | 8.21E-11 | 42.7934 |
| rs4324076 | 15 | 51510868 | -0.0201 | 0.0022 | C | 9.14E-20 | 83.47314 |
| rs60616569 | 15 | 78271261 | -0.0178 | 0.0028 | A | 1.70E-10 | 40.41327 |
| rs2047824 | 15 | 69609456 | -0.0254 | 0.0023 | C | 5.25E-29 | 121.9584 |
| rs139974673 | 15 | 44027885 | 0.1002 | 0.0079 | C | 1.58E-36 | 160.8723 |
| rs41278174 | 16 | 16259596 | 0.0501 | 0.0076 | A | 4.26E-11 | 43.45585 |
| rs34042070 | 16 | 72101525 | 0.0167 | 0.0027 | G | 3.80E-10 | 38.25652 |
| rs12933858 | 16 | 81566121 | 0.0173 | 0.0023 | T | 5.55E-14 | 56.57656 |
| rs73536752 | 16 | 47904346 | -0.0335 | 0.0061 | T | 4.14E-08 | 30.1599 |
| rs12922549 | 16 | 54451747 | -0.0221 | 0.003 | T | 1.30E-13 | 54.26778 |
| rs1858800 | 16 | 73024276 | 0.0266 | 0.0024 | T | 2.98E-28 | 122.8403 |
| rs164751 | 16 | 89707636 | -0.0188 | 0.0025 | T | 1.26E-13 | 56.5504 |
| rs12918968 | 16 | 88520452 | -0.0331 | 0.0022 | C | 1.77E-50 | 226.3657 |
| rs4790873 | 17 | 1994966 | -0.019 | 0.0025 | T | 4.94E-14 | 57.76 |
| rs6503506 | 17 | 37524653 | 0.0265 | 0.0026 | A | 1.08E-24 | 103.8831 |
| rs12953299 | 17 | 17726648 | -0.0181 | 0.0024 | G | 1.19E-13 | 56.87674 |
| rs228779 | 17 | 42091213 | -0.0181 | 0.0032 | G | 2.33E-08 | 31.99316 |
| rs77542162 | 17 | 67081278 | -0.0894 | 0.0083 | G | 3.89E-27 | 116.0163 |
| rs9895661 | 17 | 59456589 | -0.0256 | 0.0027 | T | 2.84E-21 | 89.89849 |
| rs73342502 | 17 | 6612900 | 0.0329 | 0.0052 | C | 3.05E-10 | 40.02996 |
| rs755736 | 17 | 47891904 | 0.0165 | 0.0026 | G | 2.10E-10 | 40.27367 |
| rs11078597 | 17 | 1618363 | 0.0446 | 0.0028 | C | 2.93E-57 | 253.7194 |
| rs35331358 | 18 | 60203140 | 0.0179 | 0.0027 | A | 7.25E-11 | 43.95199 |
| rs73001065 | 19 | 19460541 | 0.0349 | 0.0048 | C | 4.20E-13 | 52.86502 |
| rs1672991 | 19 | 35556659 | 0.0666 | 0.0043 | G | 1.82E-53 | 239.8897 |
| rs75702986 | 19 | 35566151 | 0.0361 | 0.0031 | A | 1.33E-30 | 135.6098 |
| rs117080418 | 19 | 50025208 | -0.099 | 0.0126 | A | 4.87E-15 | 61.73469 |
| rs117896857 | 19 | 3111094 | -0.0553 | 0.0076 | T | 3.53E-13 | 52.94477 |
| rs11881404 | 19 | 36021193 | -0.0176 | 0.0023 | G | 2.62E-14 | 58.55577 |
| rs11085015 | 19 | 3369572 | -0.0181 | 0.0032 | G | 2.11E-08 | 31.99316 |
| rs12982234 | 19 | 38586434 | -0.0582 | 0.0064 | T | 7.28E-20 | 82.69629 |
| rs149807892 | 19 | 50159756 | 0.0659 | 0.01 | T | 5.38E-11 | 43.4281 |
| rs34944502 | 19 | 3121510 | 0.0427 | 0.0027 | GC | 1.83E-54 | 250.1084 |
| rs2335534 | 19 | 50014977 | -0.0358 | 0.0031 | A | 5.40E-30 | 133.3652 |
| rs35118755 | 19 | 52172672 | 0.023 | 0.0035 | T | 4.86E-11 | 43.18367 |
| rs3091842 | 20 | 39344272 | 0.094 | 0.0062 | A | 1.85E-51 | 229.8647 |
| rs17216707 | 20 | 52732362 | -0.0581 | 0.003 | C | 3.83E-82 | 375.0678 |
| rs11086449 | 20 | 52713520 | -0.017 | 0.003 | AG | 1.77E-08 | 32.11111 |
| rs6073257 | 20 | 42561422 | 0.0133 | 0.0023 | T | 7.24E-09 | 33.43856 |
| rs11546155 | 20 | 33451148 | -0.0174 | 0.0031 | A | 2.35E-08 | 31.50468 |
| rs73075609 | 20 | 5580789 | 0.056 | 0.0076 | T | 2.42E-13 | 54.29363 |
| rs2762943 | 20 | 52790786 | 0.0505 | 0.0046 | G | 1.56E-27 | 120.5222 |
| rs928760 | 21 | 35890958 | -0.0181 | 0.0023 | T | 1.22E-14 | 61.93006 |
| rs12626330 | 21 | 37835982 | -0.0129 | 0.0022 | G | 6.18E-09 | 34.38223 |
| rs5997623 | 22 | 30756907 | 0.0147 | 0.0025 | A | 3.51E-09 | 34.5744 |
| rs2017188 | 22 | 24994708 | 0.0146 | 0.0023 | C | 3.87E-10 | 40.2949 |
| rs143193015 | 22 | 43110751 | 0.0228 | 0.0038 | GA | 2.30E-09 | 36 |

**Supplementary Table 5. 68 SNPs associated with Vitamin C.**

| **SNP** | **chre** | **pos** | **beta** | **se** | **effect_allele** | **pval** | **F** |
| --- | --- | --- | --- | --- | --- | --- | --- |
| rs147412044 | 1 | 1.68E+08 | -0.8188 | 0.1311 | T | 4.26E-10 | 39.00769 |
| rs115757213 | 1 | 2.12E+08 | -0.3982 | 0.0725 | G | 3.93E-08 | 30.16661 |
| rs145396741 | 1 | 2.26E+08 | -0.3569 | 0.0611 | A | 5.20E-09 | 34.12013 |
| rs77503249 | 1 | 2.35E+08 | -0.3558 | 0.052 | A | 7.92E-12 | 46.81717 |
| rs116716302 | 2 | 1.41E+08 | -0.461 | 0.0784 | T | 4.15E-09 | 34.5756 |
| rs146985384 | 2 | 1.42E+08 | -0.7361 | 0.1332 | G | 3.25E-08 | 30.53973 |
| rs147589962 | 2 | 1.86E+08 | -0.426 | 0.0706 | C | 1.62E-09 | 36.40909 |
| rs114637089 | 2 | 48801442 | -0.3821 | 0.0696 | A | 4.03E-08 | 30.13947 |
| rs73995784 | 2 | 2.36E+08 | -0.4812 | 0.0785 | G | 8.70E-10 | 37.57612 |
| rs150674662 | 2 | 1.23E+08 | -0.3976 | 0.0704 | A | 1.60E-08 | 31.89682 |
| rs12490747 | 3 | 9092890 | -0.2677 | 0.0484 | A | 3.19E-08 | 30.59187 |
| rs71323476 | 3 | 1.17E+08 | -0.6054 | 0.1104 | T | 4.13E-08 | 30.07092 |
| rs116768603 | 3 | 1348020 | -0.3479 | 0.0507 | G | 7.14E-12 | 47.08612 |
| rs4859019 | 3 | 88440502 | -0.4399 | 0.0701 | C | 3.44E-10 | 39.37965 |
| rs115504365 | 3 | 1.37E+08 | -0.5922 | 0.1041 | A | 1.26E-08 | 32.362 |
| rs2222837 | 3 | 1.6E+08 | -0.4193 | 0.0769 | A | 4.88E-08 | 29.73015 |
| rs115036356 | 3 | 13191877 | -0.4144 | 0.0744 | A | 2.55E-08 | 31.0237 |
| rs149277200 | 3 | 1.84E+08 | -0.5132 | 0.0861 | G | 2.53E-09 | 35.52769 |
| rs141109644 | 3 | 1.96E+08 | -0.3335 | 0.0604 | G | 3.39E-08 | 30.48722 |
| rs73122527 | 4 | 27134001 | -0.5183 | 0.0863 | A | 1.94E-09 | 36.06956 |
| rs151079977 | 4 | 95995355 | -0.3436 | 0.0593 | G | 6.76E-09 | 33.57352 |
| rs116058454 | 4 | 82799684 | -0.7361 | 0.1332 | A | 3.25E-08 | 30.53973 |
| rs147393151 | 4 | 1.38E+08 | -0.5488 | 0.0938 | T | 4.91E-09 | 34.23123 |
| rs202139829 | 4 | 1.45E+08 | -0.2837 | 0.052 | A | 4.90E-08 | 29.76542 |
| rs62389352 | 5 | 1.63E+08 | -0.6152 | 0.0682 | T | 1.95E-19 | 81.36992 |
| rs111266167 | 5 | 1.33E+08 | -0.4258 | 0.0761 | T | 2.25E-08 | 31.30704 |
| rs145489384 | 5 | 92700914 | -0.2956 | 0.0523 | G | 1.59E-08 | 31.94519 |
| rs116106286 | 5 | 1.72E+08 | -0.4276 | 0.0727 | A | 4.08E-09 | 34.59446 |
| rs6911990 | 6 | 1.53E+08 | -0.4612 | 0.0768 | C | 1.92E-09 | 36.06253 |
| rs16889083 | 6 | 36729068 | -0.2534 | 0.0396 | T | 1.52E-10 | 40.94707 |
| rs239825 | 6 | 54773200 | -0.4749 | 0.08 | C | 2.95E-09 | 35.23906 |
| rs146857350 | 6 | 1.13E+08 | -0.5956 | 0.079 | A | 4.77E-14 | 56.84015 |
| rs116967294 | 6 | 95056239 | -0.7594 | 0.1334 | C | 1.26E-08 | 32.40631 |
| rs76715584 | 6 | 1.53E+08 | -0.2278 | 0.0379 | T | 1.86E-09 | 36.12676 |
| rs144580260 | 7 | 48052891 | -0.519 | 0.0859 | A | 1.54E-09 | 36.50467 |
| rs143142132 | 7 | 12082686 | -0.8303 | 0.1305 | T | 1.96E-10 | 40.4808 |
| rs181246816 | 8 | 1278957 | -0.2302 | 0.0398 | T | 7.38E-09 | 33.45373 |
| rs77222063 | 8 | 21773577 | -0.8769 | 0.1134 | G | 1.02E-14 | 59.79626 |
| rs35664127 | 9 | 73622580 | -0.411 | 0.0702 | A | 4.90E-09 | 34.27752 |
| rs139991506 | 9 | 23704505 | -0.8069 | 0.1311 | G | 7.42E-10 | 37.8821 |
| rs190279780 | 9 | 8949161 | -0.416 | 0.0687 | A | 1.44E-09 | 36.66681 |
| rs116559716 | 9 | 98210170 | -0.5459 | 0.0968 | A | 1.69E-08 | 31.80354 |
| rs35248101 | 10 | 13171617 | -0.2336 | 0.0409 | C | 1.16E-08 | 32.62113 |
| rs138662264 | 10 | 1.01E+08 | -0.8716 | 0.1062 | C | 2.31E-16 | 67.35741 |
| rs141694582 | 10 | 30833134 | -0.3648 | 0.0649 | A | 1.93E-08 | 31.59514 |
| rs77992796 | 10 | 1.29E+08 | -0.4948 | 0.0831 | A | 2.66E-09 | 35.45335 |
| rs3026057 | 10 | 1.19E+08 | -0.8949 | 0.1352 | A | 3.60E-11 | 43.81226 |
| rs118027537 | 12 | 16085254 | -0.8831 | 0.1303 | C | 1.22E-11 | 45.93365 |
| rs77459185 | 12 | 77206531 | -0.3061 | 0.0454 | G | 1.54E-11 | 45.45848 |
| rs77820382 | 12 | 1.12E+08 | -0.5747 | 0.0767 | A | 6.84E-14 | 56.14249 |
| rs145119123 | 13 | 1.08E+08 | -0.2923 | 0.0526 | T | 2.81E-08 | 30.88063 |
| rs117042417 | 13 | 66201226 | -0.4897 | 0.0789 | A | 5.34E-10 | 38.52177 |
| rs142070515 | 13 | 92501248 | -0.6196 | 0.1003 | G | 6.49E-10 | 38.16111 |
| rs146783469 | 14 | 60859899 | -0.7732 | 0.1311 | G | 3.64E-09 | 34.7839 |
| rs143302471 | 16 | 2895094 | -0.5206 | 0.086 | A | 1.39E-09 | 36.64472 |
| rs141568393 | 16 | 22944021 | -0.4512 | 0.0805 | G | 2.08E-08 | 31.41568 |
| rs373008011 | 16 | 84127498 | -0.5831 | 0.0904 | T | 1.09E-10 | 41.60535 |
| rs17637408 | 16 | 26295044 | -0.2981 | 0.0527 | C | 1.49E-08 | 31.99652 |
| rs1399998 | 16 | 27163332 | -0.753 | 0.1307 | C | 8.36E-09 | 33.19241 |
| rs117193023 | 18 | 5643874 | -0.355 | 0.0636 | T | 2.35E-08 | 31.15606 |
| rs80148899 | 18 | 22668598 | -0.9042 | 0.1051 | T | 7.71E-18 | 74.01565 |
| rs12454633 | 18 | 40494893 | -0.3071 | 0.0532 | A | 7.89E-09 | 33.32241 |
| rs12462583 | 19 | 38905792 | -0.2106 | 0.0358 | A | 4.24E-09 | 34.60594 |
| rs6030549 | 20 | 41519220 | -0.645 | 0.1114 | C | 7.00E-09 | 33.52348 |
| rs187748425 | 21 | 40558118 | -0.5082 | 0.071 | A | 8.40E-13 | 51.23333 |
| rs147668949 | 21 | 28314693 | -0.7291 | 0.131 | A | 2.61E-08 | 30.97645 |
| rs149503879 | 21 | 45271053 | -0.5354 | 0.0866 | A | 6.31E-10 | 38.22266 |
| rs113757848 | 22 | 34545881 | -0.538 | 0.0897 | A | 2.01E-09 | 35.97325 |

**Supplementary Table 6. 117 SNPs associated with Vitamin D.**

| **SNP** | **chr** | **pos** | **beta** | **se** | **effect_allele** | **pval** | **F** |
| --- | --- | --- | --- | --- | --- | --- | --- |
| rs11207969 | 1 | 62911751 | 0.02094 | 0.002127 | G | 7.14E-23 | 96.94293 |
| rs11264361 | 1 | 1.55E+08 | 0.017488 | 0.002341 | G | 7.97E-14 | 55.81143 |
| rs61747728 | 1 | 1.8E+08 | 0.030306 | 0.005269 | T | 8.83E-09 | 33.08366 |
| rs2807834 | 1 | 2.21E+08 | -0.01506 | 0.002187 | G | 5.66E-12 | 47.44429 |
| rs512083 | 1 | 46027355 | 0.012217 | 0.002043 | C | 2.23E-09 | 35.76565 |
| rs6672758 | 1 | 2.3E+08 | 0.016248 | 0.002555 | T | 2.04E-10 | 40.42698 |
| rs2494429 | 1 | 2339395 | -0.01485 | 0.002673 | G | 2.80E-08 | 30.83954 |
| rs1343776 | 1 | 41757718 | 0.018076 | 0.00245 | A | 1.62E-13 | 54.42305 |
| rs7528419 | 1 | 1.1E+08 | 0.021539 | 0.002432 | G | 8.17E-19 | 78.45939 |
| rs115288876 | 1 | 1.52E+08 | 0.078807 | 0.004982 | A | 2.36E-56 | 250.1878 |
| rs35823191 | 1 | 17560123 | -0.02326 | 0.002141 | C | 1.65E-27 | 118.0957 |
| rs61813875 | 1 | 1.53E+08 | 0.082129 | 0.006589 | G | 1.16E-35 | 155.377 |
| rs1042034 | 2 | 21225281 | -0.01513 | 0.0025 | T | 1.45E-09 | 36.59946 |
| rs1260326 | 2 | 27730940 | 0.019719 | 0.002074 | C | 1.96E-21 | 90.382 |
| rs35270497 | 2 | 38259872 | 0.015672 | 0.002682 | T | 5.08E-09 | 34.15937 |
| rs7569755 | 2 | 1.19E+08 | 0.01364 | 0.002256 | A | 1.49E-09 | 36.54421 |
| rs2710651 | 2 | 63166379 | -0.01159 | 0.002035 | A | 1.23E-08 | 32.44061 |
| rs3732220 | 2 | 2.35E+08 | -0.04784 | 0.003633 | A | 1.31E-39 | 173.4378 |
| rs727857 | 2 | 58981967 | -0.01205 | 0.002099 | A | 9.27E-09 | 32.98904 |
| rs7580771 | 2 | 1.01E+08 | -0.01656 | 0.002665 | T | 5.15E-10 | 38.61821 |
| rs1047891 | 2 | 2.12E+08 | -0.0134 | 0.00218 | A | 7.96E-10 | 37.76845 |
| rs7652808 | 3 | 85603643 | -0.02129 | 0.002126 | G | 1.36E-23 | 100.2297 |
| rs9847248 | 3 | 18804655 | -0.01231 | 0.002245 | A | 4.19E-08 | 30.05959 |
| rs13076508 | 3 | 52407805 | 0.025054 | 0.00451 | C | 2.78E-08 | 30.85642 |
| rs1128535 | 3 | 49866392 | 0.016413 | 0.002029 | T | 6.06E-16 | 65.41671 |
| rs6438900 | 3 | 1.25E+08 | 0.015049 | 0.00234 | G | 1.27E-10 | 41.34713 |
| rs34186890 | 3 | 1.42E+08 | -0.01569 | 0.002318 | G | 1.33E-11 | 45.77335 |
| rs1949633 | 3 | 1.54E+08 | 0.011416 | 0.002086 | C | 4.45E-08 | 29.9406 |
| rs6834488 | 4 | 88178919 | -0.01445 | 0.002059 | T | 2.26E-12 | 49.24664 |
| rs13108245 | 4 | 57790205 | -0.01222 | 0.002086 | G | 4.63E-09 | 34.33793 |
| rs3114045 | 4 | 1E+08 | -0.02217 | 0.00298 | C | 1.00E-13 | 55.36311 |
| rs78649910 | 4 | 3482213 | -0.01914 | 0.003322 | A | 8.33E-09 | 33.19553 |
| rs4348160 | 4 | 70017531 | -0.02584 | 0.002163 | G | 6.62E-33 | 142.7657 |
| rs4147536 | 4 | 1E+08 | -0.0148 | 0.00249 | C | 2.76E-09 | 35.34462 |
| rs4364259 | 4 | 15892159 | 0.017241 | 0.002567 | A | 1.86E-11 | 45.11742 |
| rs71599974 | 4 | 71765339 | 0.025738 | 0.002862 | G | 2.39E-19 | 80.88318 |
| rs12501515 | 4 | 72592838 | -0.07896 | 0.002069 | A | ####### | 1455.881 |
| rs11726886 | 4 | 72822599 | -0.05367 | 0.002255 | A | ####### | 566.6014 |
| rs7712001 | 5 | 1.48E+08 | 0.011939 | 0.002062 | G | 7.05E-09 | 33.51934 |
| rs986649 | 5 | 1.19E+08 | 0.012864 | 0.002178 | G | 3.51E-09 | 34.87966 |
| rs17207784 | 6 | 22768668 | -0.01349 | 0.002171 | C | 5.14E-10 | 38.62205 |
| rs1321247 | 6 | 25662873 | -0.02218 | 0.003366 | T | 4.36E-11 | 43.44459 |
| rs12153819 | 6 | 83773049 | -0.01782 | 0.003091 | T | 8.16E-09 | 33.23706 |
| rs742493 | 6 | 40998167 | 0.018353 | 0.003206 | C | 1.04E-08 | 32.76866 |
| rs2245133 | 6 | 1.32E+08 | -0.02129 | 0.00274 | C | 7.80E-15 | 60.38412 |
| rs9375037 | 6 | 1.22E+08 | 0.011706 | 0.002054 | C | 1.21E-08 | 32.47218 |
| rs1858889 | 7 | 1.07E+08 | 0.013451 | 0.002031 | C | 3.49E-11 | 43.87982 |
| rs2595644 | 7 | 43980540 | -0.01226 | 0.002097 | T | 4.97E-09 | 34.19978 |
| rs7784802 | 7 | 64015379 | 0.01332 | 0.002116 | T | 3.06E-10 | 39.63814 |
| rs10277163 | 7 | 21569089 | -0.01433 | 0.002351 | G | 1.08E-09 | 37.18058 |
| rs804281 | 8 | 11611865 | 0.015895 | 0.00206 | G | 1.20E-14 | 59.53869 |
| rs1384687 | 8 | 61525963 | -0.01687 | 0.002997 | A | 1.82E-08 | 31.67397 |
| rs12056768 | 8 | 1.17E+08 | -0.0232 | 0.002064 | G | 2.65E-29 | 126.2948 |
| rs34726834 | 8 | 25889606 | 0.014013 | 0.002349 | T | 2.42E-09 | 35.60013 |
| rs9409266 | 9 | 1.26E+08 | -0.01678 | 0.002947 | A | 1.24E-08 | 32.41927 |
| rs13294734 | 9 | 80710910 | 0.012568 | 0.002058 | T | 1.02E-09 | 37.28082 |
| rs635634 | 9 | 1.36E+08 | -0.01505 | 0.002604 | T | 7.55E-09 | 33.38765 |
| rs11791258 | 9 | 1.08E+08 | 0.014081 | 0.002581 | A | 4.85E-08 | 29.77421 |
| rs2398113 | 10 | 10076429 | -0.01176 | 0.002058 | G | 1.10E-08 | 32.65735 |
| rs12775091 | 10 | 91524012 | 0.015562 | 0.002477 | T | 3.33E-10 | 39.47228 |
| rs2297991 | 10 | 1.14E+08 | 0.012755 | 0.002256 | C | 1.57E-08 | 31.97032 |
| rs77532868 | 10 | 88081438 | 0.025956 | 0.004563 | T | 1.28E-08 | 32.35658 |
| rs144965707 | 11 | 14059511 | -0.03481 | 0.004218 | A | 1.52E-16 | 68.13918 |
| rs1627043 | 11 | 71110175 | -0.04864 | 0.005661 | C | 8.49E-18 | 73.83543 |
| rs2847500 | 11 | 1.2E+08 | -0.02255 | 0.003087 | A | 2.77E-13 | 53.36313 |
| rs17473257 | 11 | 14283186 | -0.06114 | 0.0078 | A | 4.59E-15 | 61.42892 |
| rs117300835 | 11 | 15118975 | -0.33499 | 0.00886 | A | ####### | 1429.481 |
| rs2511279 | 11 | 71130419 | 0.098172 | 0.005208 | G | 2.98E-79 | 355.2964 |
| rs3829251 | 11 | 71194559 | -0.11445 | 0.002981 | A | ####### | 1474.547 |
| rs11023159 | 11 | 14262063 | 0.048212 | 0.005725 | C | 3.73E-17 | 70.91585 |
| rs733454 | 11 | 76477721 | 0.018855 | 0.0034 | T | 2.93E-08 | 30.75174 |
| rs111515741 | 11 | 14370944 | -0.04874 | 0.00779 | A | 3.95E-10 | 39.13658 |
| rs12283049 | 11 | 14690192 | -0.05646 | 0.002406 | G | ####### | 550.5384 |
| rs11600054 | 11 | 14690511 | 0.068175 | 0.010148 | A | 1.84E-11 | 45.13388 |
| rs964184 | 11 | 1.17E+08 | 0.040685 | 0.002989 | C | 3.50E-42 | 185.2258 |
| rs61887421 | 11 | 70949673 | -0.03673 | 0.005978 | C | 8.05E-10 | 37.74872 |
| rs7955128 | 12 | 38684121 | 0.013062 | 0.002039 | T | 1.48E-10 | 41.05371 |
| rs1038165 | 12 | 68665940 | 0.011515 | 0.002056 | T | 2.15E-08 | 31.35824 |
| rs73413596 | 12 | 1.12E+08 | 0.022347 | 0.003889 | C | 9.15E-09 | 33.01355 |
| rs28435470 | 12 | 1.33E+08 | -0.01187 | 0.002148 | A | 3.29E-08 | 30.52659 |
| rs57601828 | 12 | 93192127 | 0.011542 | 0.002082 | T | 2.96E-08 | 30.73506 |
| rs1871395 | 12 | 21352315 | -0.02037 | 0.002827 | G | 5.72E-13 | 51.94036 |
| rs2171427 | 12 | 24822154 | -0.01655 | 0.002817 | A | 4.26E-09 | 34.50225 |
| rs10859995 | 12 | 96375682 | -0.04363 | 0.002055 | C | ####### | 450.8977 |
| rs4580037 | 13 | 55702646 | -0.01356 | 0.002251 | C | 1.68E-09 | 36.30967 |
| rs8018720 | 14 | 39556185 | -0.0345 | 0.002661 | C | 1.94E-38 | 168.0884 |
| rs2756119 | 14 | 1.04E+08 | 0.012143 | 0.00211 | A | 8.71E-09 | 33.10938 |
| rs142004400 | 14 | 50829560 | -0.031 | 0.005596 | C | 3.01E-08 | 30.69897 |
| rs1532085 | 15 | 58683366 | 0.025281 | 0.002086 | G | 8.60E-34 | 146.8183 |
| rs1800588 | 15 | 58723675 | -0.0305 | 0.002469 | T | 4.73E-35 | 152.5825 |
| rs62007299 | 15 | 77711719 | -0.01242 | 0.002244 | A | 3.12E-08 | 30.63248 |
| rs325393 | 15 | 1E+08 | -0.01365 | 0.002277 | T | 2.03E-09 | 35.94278 |
| rs12324720 | 15 | 64092140 | -0.01492 | 0.002675 | A | 2.45E-08 | 31.10359 |
| rs1684600 | 16 | 4594671 | -0.01253 | 0.002217 | T | 1.59E-08 | 31.93767 |
| rs11542462 | 16 | 82033810 | -0.02478 | 0.002983 | A | 9.72E-17 | 69.02534 |
| rs77924615 | 16 | 20392332 | -0.01525 | 0.00259 | A | 3.94E-09 | 34.65246 |
| rs11076175 | 16 | 57006378 | 0.022903 | 0.00267 | G | 9.64E-18 | 73.58399 |
| rs11867297 | 17 | 66433493 | 0.013543 | 0.002095 | T | 1.01E-10 | 41.80857 |
| rs61698755 | 17 | 79257880 | -0.01147 | 0.002051 | C | 2.25E-08 | 31.26285 |
| rs9946771 | 18 | 28918628 | -0.0234 | 0.004077 | T | 9.47E-09 | 32.94705 |
| rs2037511 | 18 | 61366207 | 0.017662 | 0.002727 | A | 9.41E-11 | 41.93989 |
| rs77960347 | 18 | 47109955 | -0.05257 | 0.00906 | G | 6.53E-09 | 33.66939 |
| rs10438978 | 18 | 47158186 | -0.01722 | 0.002644 | C | 7.34E-11 | 42.42665 |
| rs1048328 | 19 | 51527364 | 0.03135 | 0.003744 | A | 5.58E-17 | 70.12143 |
| rs142158911 | 19 | 11190534 | 0.026284 | 0.003234 | A | 4.43E-16 | 66.03579 |
| rs12462826 | 19 | 11955767 | -0.01321 | 0.002115 | A | 4.18E-10 | 39.02572 |
| rs4420638 | 19 | 45422946 | -0.0193 | 0.002659 | G | 3.95E-13 | 52.66718 |
| rs8107974 | 19 | 19388500 | 0.035567 | 0.003823 | T | 1.36E-20 | 86.54748 |
| rs62129966 | 19 | 48374950 | 0.061164 | 0.002764 | A | ####### | 489.773 |
| rs1841850 | 20 | 52718179 | 0.03044 | 0.003165 | C | 6.73E-22 | 92.50225 |
| rs8121940 | 20 | 52742306 | -0.04356 | 0.002549 | G | 1.77E-65 | 292.0565 |
| rs6129648 | 20 | 39231118 | 0.014063 | 0.002106 | G | 2.44E-11 | 44.57896 |
| rs290400 | 20 | 52698179 | -0.0131 | 0.002163 | A | 1.41E-09 | 36.64866 |
| rs2229742 | 21 | 16339172 | -0.02498 | 0.003314 | C | 4.75E-14 | 56.83095 |
| rs138335 | 22 | 41227086 | -0.01377 | 0.002151 | G | 1.56E-10 | 40.95369 |
| rs2074735 | 22 | 31535872 | 0.029267 | 0.00412 | C | 1.22E-12 | 50.46068 |
| rs5770794 | 22 | 50880781 | -0.01331 | 0.002211 | T | 1.74E-09 | 36.24743 |
